# Assessing species diversity of Coral Triangle artisanal fisheries: A DNA barcode reference library for the shore fishes retailed at Ambon harbor (Indonesia)

**DOI:** 10.1002/ece3.6128

**Published:** 2020-03-06

**Authors:** Gino Limmon, Erwan Delrieu‐Trottin, Jesaya Patikawa, Frederik Rijoly, Hadi Dahruddin, Frédéric Busson, Dirk Steinke, Nicolas Hubert

**Affiliations:** ^1^ Pusat Kemaritiman dan Kelautan Universitas Pattimura (Maritime and Marine Science Center of Excellence) Ambon Indonesia; ^2^ Institut de Recherche pour le Développement UMR 226 ISEM (UM‐CNRS‐IRD‐EPHE) Montpellier France; ^3^ Museum für Naturkunde Leibniz‐Institut für Evolutions‐und Biodiversitätsforschung an der Humboldt‐Universität zu Berlin Berlin Germany; ^4^ Division of Zoology Research Center for Biology Indonesian Institute of Sciences (LIPI) Cibinong Indonesia; ^5^ UMR 7208 BOREA (MNHN‐CNRS‐UPMC‐IRD‐UCBN) Muséum National d’Histoire Naturelle Paris France; ^6^ Department of Integrative Biology Centre for Biodiversity Genomics University of Guelph Guelph ON Canada

**Keywords:** fisheries assessment, reference library, species delimitation, species diagnostic, Wallacea

## Abstract

The Coral Triangle (CT), a region spanning across Indonesia and Philippines, is home to about 4,350 marine fish species and is among the world's most emblematic regions in terms of conservation. Threatened by overfishing and oceans warming, the CT fisheries have faced drastic declines over the last decades. Usually monitored through a biomass‐based approach, fisheries trends have rarely been characterized at the species level due to the high number of taxa involved and the difficulty to accurately and routinely identify individuals to the species level. Biomass, however, is a poor proxy of species richness, and automated methods of species identification are required to move beyond biomass‐based approaches. Recent meta‐analyses have demonstrated that species richness peaks at intermediary levels of biomass. Consequently, preserving biomass is not equal to preserving biodiversity. We present the results of a survey to estimate the shore fish diversity retailed at the harbor of Ambon Island, an island located at the center of the CT that display exceptionally high biomass despite high levels of threat, while building a DNA barcode reference library of CT shore fishes targeted by artisanal fisheries. We sampled 1,187 specimens and successfully barcoded 696 of the 760 selected specimens that represent 202 species. Our results show that DNA barcodes were effective in capturing species boundaries for 96% of the species examined, which opens new perspectives for the routine monitoring of the CT fisheries.

## Graphical abstract

The Coral Triangle is home of the highest marine biodiversity. Artisanal fisheries are extremely important in the Coral Triangle. Fish species are poorly known and hardly identifiable. We propose a DNA barcode reference library for the fish harvested by the artisanal fisheries in Ambon Island.

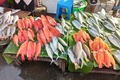

## 1 INTRODUCTION

Located at the boundary between the Indian and Pacific Ocean, the Coral Triangle (CT) is home to some 4,350 marine fish species (Froese & Pauly, 2014). It encompasses both the Philippine and the Indonesian archipelagoes, constituting the largest diversity anomaly in the world's oceans with local species richness peaking at about 2,500 fish species per 5° × 5° grid cell. This rich biodiversity is decreasing rapidly (Bellwood & Meyer, 2009; Gaboriau, Leprieur, Mouillot, & Hubert, 2018; Pellissier et al., 2014) as a consequence of ocean warming (Garciá Molinos et al., 2016), overfishing, habitat degradation, and other unsustainable human activities. The future of the CT's biodiversity has received increased attention over the two last decades (Allen, 2008; Cinner et al., 2016; Roberts, 2002). Our ability to anticipate and potentially mitigate biodiversity loss is of prime importance and will serve as an example for our ability to preserve an exceptionally rich area. Species loss in the CT is not only a conservation concern but also poses a food security challenge similar to one faced by most tropical and biodiversity‐rich countries (Lal, 2004; Lobell et al., 2008; Schmidhuber & Tubiello, 2008). In a region where fish consumption accounts for more than 20% of the daily animal protein intake and fish supply largely relies on one of the largest fisheries worldwide (FAO, 2018), sustainable management of fish resources is of broad concern. The issue is amplified by the global decline of marine fish stocks, which the current rate, questions both the sustainability of ocean resource harvesting, and the resilience of marine ecosystems (Hughes et al., 2003; Tittensor et al., 2010).

Most large‐scale fisheries studies for the Indo‐Pacific ocean have focused on linking biomass through space and/or time to varying parameters affecting them (Cinner, Graham, Huchery, & Macneil, 2013; Cinner et al., 2016; Maire et al., 2016). In ecosystems home to thousands of species and tenths of closely related and morphologically similar species, biomass represents a straightforward shortcut to address ecosystem functioning and fisheries dynamics. It can be used to monitor fisheries through time and detect potential stock collapses (Cinner et al., 2016). While this approach allows to document large‐scale fisheries trends and to identify regions where those jeopardize food security, it is of limited value for the exploration of ecological dynamics driving species coexistence and ecosystem resilience (Chase & Leibold, 2002; Gravel et al., 2011; Massol et al., 2011; Yachi & Loreau, 1999). In addition, biomass estimates are usually derived from fisheries statistics or underwater visual census through allometric length–weight relationships, further converted into units of mass per area, a proxy that only account for a fraction of the coral reef biodiversity. This level of granularity and the increasing consumer demand for better food traceability requires reliable and routine species level identification of fish products.

DNA barcoding, the use of a 650 base pair sequence of the mitochondrial Cytochrome Oxidase I gene as an internal species tag (Hebert, Cywinska, Ball, & Waard, 2003), provides the necessary ability to assign unknown samples to known species independent of the source of samples. It also offers an unprecedented level of resolution in cases where traditional morphology failed to reliably assign unknowns to knowns and thereby enables a number of applications for marine fisheries and ecosystem research such as the detection of sea food market substitutions (Chin, Adibah, Danial Hariz, & Siti Azizah, 2016; Di Pinto et al., 2015; Holmes, Steinke, & Ward, 2009; Pardo et al., 2018; Wong & Hanner, 2008), the detection of previously unrecorded species (Collet et al., 2018; Kiszka et al., 2018) or the exploration of early life stage dynamics at the species level (Hubert, Espiau, Meyer, & Planes, 2015; Kimmerling et al., 2018; Steinke, Connell, & Hebert, 2016). The utility of DNA barcoding, however, always depends on the taxonomic coverage of an associated DNA barcode reference library. Recent DNA barcoding studies of reef fish diversity in the Indo‐Pacific Ocean unraveled historical taxonomic conflicts, discovered cryptic or unrecognized diversity, and confirmed the need for more complete reference libraries for this region (Durand, Hubert, Shen, & Borsa, 2017; Hubert et al., 2012, 2017; Randall & Victor, 2015; Steinke, Zemlak, & Hebert, 2009; Winterbottom, Hanner, Burridge, & Zur, 2014).

In order to determine the fish diversity harvested by artisanal fisheries in the CT, we set out to build a DNA barcode inventory of the shore fish species retailed at Ambon Island. This approach not only accounts for potential cryptic diversity but also helps to establish a sustainable resource for fisheries monitoring and food traceability. Ambon Island is located in the centre of the CT and has been identified as one of a few bright spots in the Indo‐Pacific where biomass is still higher than expected especially when considering overall environmental conditions and socioeconomic drivers of the region (Cinner et al., 2016). Ambon Island can serve as a model for artisanal fisheries in less perturbed parts of the CT, and the diversity of landed fish determined by our study is expected to provide valuable information on fisheries trends elsewhere.

## 2 MATERIALS AND METHODS

### 2.1 Sampling, morphological identification, and acquisition of DNA barcodes

**Figure 1:**
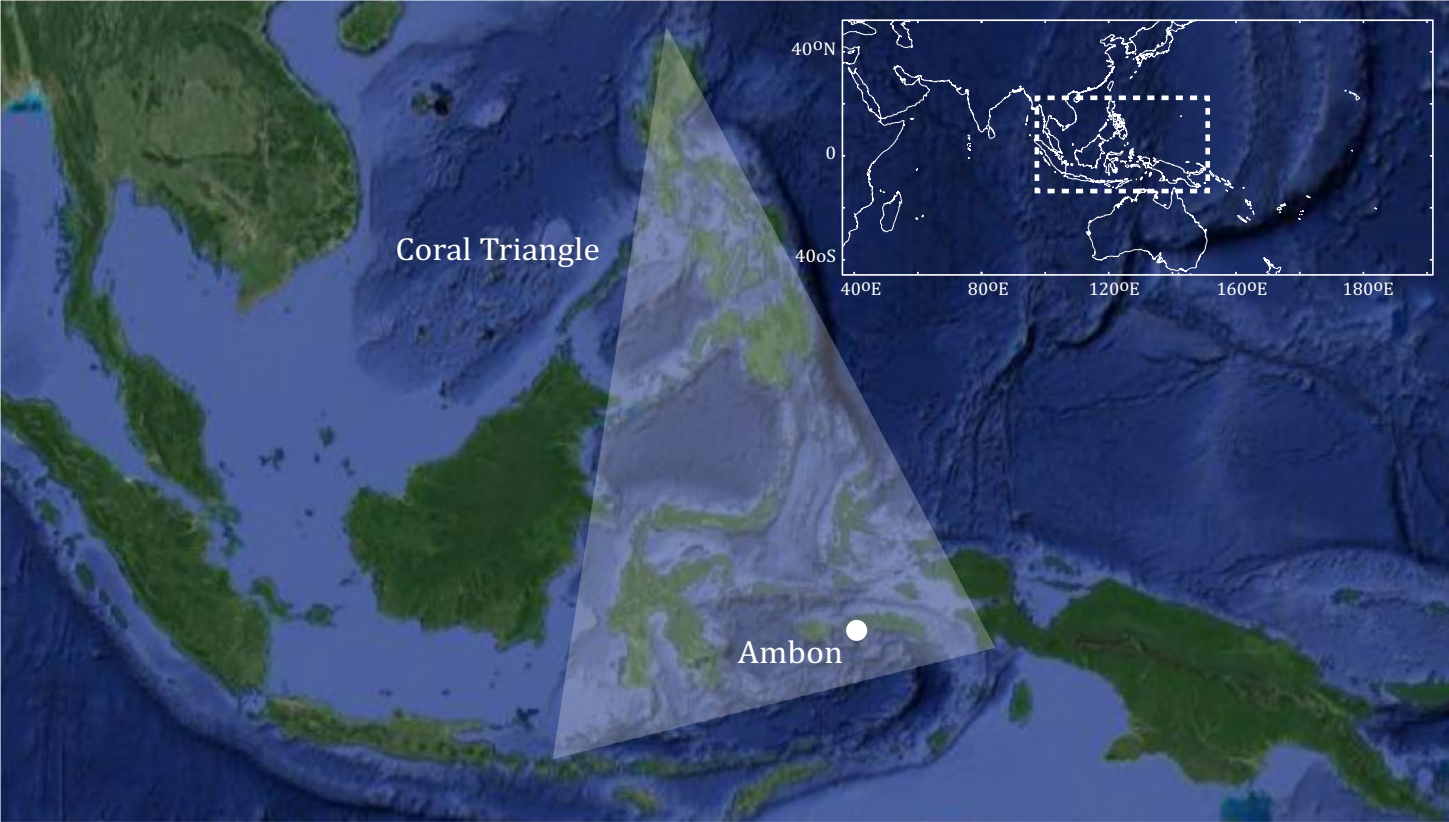
Map of the Coral Triangle and Ambon Island

A total of 1,187 specimens were collected at Ambon harbor between March and December 2016 (Figure 1). Specimens were selected at the harbor based on the morphological diversity available at each time the fish stalls were visited. Provided a species was sampled on day one, the same species was sampled again if encountered later in order to build a comprehensive coverage of the taxonomic diversity retailed. This was done to ensure that closely related species, potentially requiring a more careful examination of diagnostic morphological characters, were not overlooked. Morphological identification was initially done following (Erdmann & Allen, 2012) and subsequently (postsequencing) confirmed through a Barcode Index Number (BIN) discordance report as implemented in the Barcode of Life Datasystem (BOLD—Ratnasingham & Hebert, 2007) in order to spot potential misidentification. Upon detection of discordances, secondary morphological identifications were performed using original species descriptions of the taxa under scrutiny. Specimens were photographed and individually labeled, and voucher specimens were preserved in a 5% formalin solution. A fin clip or a muscle biopsy was taken for each specimen prior to preservation and fixed in a 96% ethanol solution for further genetic analyses. Both tissue and voucher specimens were deposited at the collection of the Maritime and Marine Science Center of Excellence at the University of Pattimura.

A total of 760 specimens were selected for sequencing in order to cover as much as possible of the intraspecific genetic diversity by selecting specimens originating from different sampling events in order to validate the identification hypotheses initially produced. Genomic DNA was extracted using a Qiagen DNeasy 96 tissue extraction kit following the manufacturer's specifications. The standard 652‐bp segment from the 5' region of the cytochrome oxidase I (COI) was subsequently amplified under the following thermal conditions: Two min at 95°C; 35 cycles of 0.5 min at 94°C, 0.5 min at 52°C, and 1 min at 72°C; 10 min at 72°C; then held at 4°C. The 12.5 μl PCR reaction mixes included 6.25 μl of 10% trehalose, 2.00 μl of ultrapure water, 1.25 μl 10X PCR buffer (200 mM Tris‐HCl [pH 8.4], 500 mM KCl), 0.625 μl MgCl (50 mM), 0.125 μl of each primer cocktail (0.01 mM, using primer cocktails C_FishF1t1 and C_FishR1t1 or C_VF1LFt1 and C_VR1LRt1 (Ivanova et al. 2007), 0.062 μl of each dNTP (10 mM), 0.060 μl of Platinum^®^ Taq Polymerase (Invitrogen), and 2.0 μl of DNA template. PCR amplicons were visualized on a 1.2% agarose gel E‐Gel^®^ (Invitrogen) and bidirectionally sequenced using sequencing primers M13F or M13R and the BigDye^®^ Terminator v.3.1 Cycle Sequencing kit (Applied Biosystems, Inc.) on an ABI 3730xl capillary sequencer following manufacturer's instructions (for more detail and alternatives see Steinke, Prosser, & Hebert, 2016). Bidirectional sequences were assembled and edited using CodonCode Aligner software (CodonCode Corporation) prior to their upload.

DNA barcodes, photographs, sequences, and collection data were deposited on BOLD in the data set “DNA barcode reference library for the commercial shore fishes of Ambon Island” (https://doi.org/10.5883/DS-BMFAMB). Sequences were also submitted to GenBank, and Accession Numbers are accessible through the BOLD individual records.

### 2.2 Species delimitation and genetic distances

DNA sequence divergence was calculated using the Kimura 2‐parameter (K2P) model. The midpoint rooted Neighbor‐joining (NJ) tree of K2P distances was constructed to provide a graphic representation of the species divergence as implemented in the Sequence Analysis module of BOLD (Ratnasingham & Hebert, 2007). Sequence divergence below and above species boundaries was determined by calculating the maximum intraspecific distance and the distance to the closest phylogenetic neighbor in the data set.

Four sequence‐based methods were used for species delimitation. From now on, species identified based on traditional morphological characters will be referred to as species while species delimited by DNA sequences will be referred to as Operational Taxonomic Unit (OTU), which represent diagnosable molecular lineages (Avise, 1989; Moritz, 1994; Vogler & DeSalle, 1994). The species delimitation methods used are based on different assumptions but they all focus on the detection of transition points between mutation/drift (within species) and speciation/extinction (between species) dynamics (Hubert & Hanner, 2015). Each method is susceptible to pitfalls which is why our final delimitation scheme was based on a 50% consensus among methods in order to produce a robust delimitation (Kekkonen & Hebert, 2014; Kekkonen, Mutanen, Kaila, Nieminen, & Hebert, 2015). OTUs were delimited using the following algorithms: (a) Refined Single Linkage (RESL) as implemented in BOLD and used to produce Barcode Index Numbers (BIN) (Ratnasingham & Hebert, 2013), (b) Automatic Barcode Gap Discovery (ABGD) (Puillandre, Lambert, Brouillet, & Achaz, 2012), (c) Poisson Tree Process (PTP) in its multiple rates version as implemented in the stand‐alone software mptp_0.2.3 (Kapli et al., 2017; Zhang, Kapli, Pavlidis, & Stamatakis, 2013), and (d) General Mixed Yule‐Coalescent (GMYC) in its multiple rates version as implemented in the R package Splits 1.0‐19 (Fujisawa & Barraclough, 2013). The mPTP algorithm uses a phylogenetic tree as an input file; thus, a maximum likelihood (ML) tree was first reconstructed using RAxML (Stamatakis, Ludwig, & Meier, 2005) based on a GTR + Γ substitution model. An ultrametric and fully resolved tree was reconstructed using a Bayesian approach implemented in BEAUti and BEAST 2.4.8 (Bouckaert et al., 2014) to be further used for OTU delimitation using the mGMYC algorithm. Two Markov chains of 50 millions each were run independently using a strict‐clock model, based on a canonical 1.2% of genetic divergence per million years (Bermingham, McCafferty, & Martin, 1997), and with a HKY + I + Γ substitution model. Trees were sampled every 10,000 states after an initial burnin period of 10 million, and both runs were combined using LogCombiner 2.4.8 (Bouckaert et al., 2014). A maximum credibility tree was constructed using TreeAnnotator 2.4.7 (Bouckaert et al., 2014). Duplicated sequences were pruned prior to the Bayesian analysis.

## 3 RESULTS

**Figure 2:**
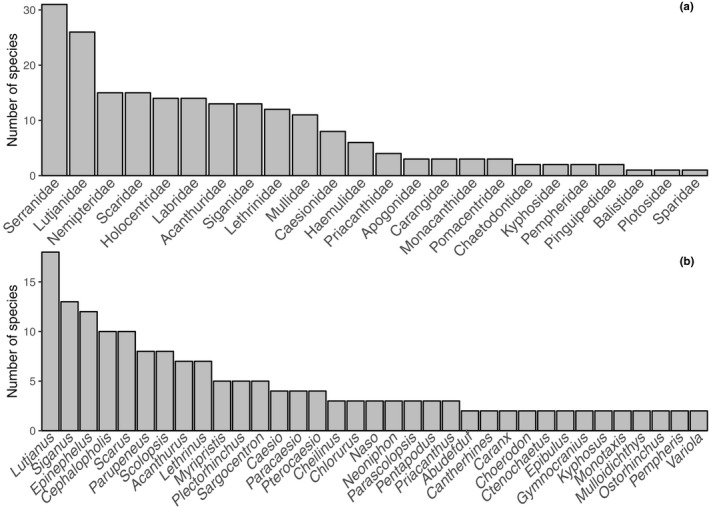
Ranking of families (a) and genera (b) according to the number of species observed is this survey

We were able to obtain 696 sequences representing 202 species, 73 genera and 24 families from 760 sampled individuals (92%). Amplification failures were randomly distributed among species, and all species were successfully sequenced. Sequence length for all barcodes was >600 bp and no codon stops were detected suggesting that these sequences correspond to functional coding regions. Of the 24 families sampled, the families Serranidae (groupers), Lutjanidae (Snappers) displayed the highest species richness with 31 and 26 species, respectively (Figure 2a). Both families were followed by eight additional families (Nemipteridae, Scarida, Holocentridae, Labridae, Acanthuridae, Siganidae, Lethrinidae, and Mullidae) with a species richness larger than ten species (Figure 2a). Of all genera obtained, the Snapper genus *Lutjanus* displayed the highest species richness with 18 species, followed by *Siganus* with 13 species, the groupers *Epinephelus* and *Cephalopholis* with 12 and 10 species, respectively (Figure 2b).

**Figure 3:**
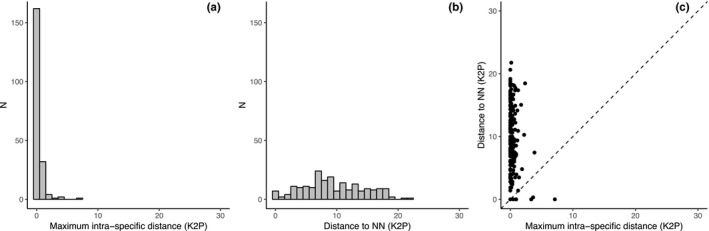
Distribution of genetic distances below and above species boundaries. (a) Distribution of maximum intraspecific distances (K2P). (b) Distribution of nearest neighbor distances (K2P). (c) Relationship between maximum intraspecific and nearest neighbor distances. Points above the diagonal line indicate species with a barcode gap

Intraspecific distances ranged from 0% to 7.12% with an average of 0.32% while interspecific distances within genus ranged from 0% to 21.83% (average 12.05%; Table 1). The distributions of the maximum intraspecific distance and the distance to the nearest neighbor overlapped (Figure 3a,b); however, nearest neighbor distances were 30‐fold higher on average than maximum intraspecific distances (Table S2). Maximum intraspecific distances exceeded nearest neighbor distance only for six species *Siganus canaliculatus* (7.12%), *Lethrinus ornatus* (3.86%), *Siganus vermiculatus* (3.64%), *Lutjanus decussatus* (3.64%), *Nemipterus furcosus* (2.36%), and *Paracaesio xanthura* (2.21%) and a barcoding gap was generally observed (Figure 3c) (Table S2 and Figure S1). By contrast, K2P distances to the nearest neighbor <2% were observed for eleven species including six cases of haplotype sharing (*Lutjanus decussatus*, *L. semicinctus*, *Siganus canaliculatus*, *S. fuscescens*, *S. guttatus*, and *S. lineatus*) and five cases of low K2P distances (*Siganus vermiculatus*, *S. corallinus*, *S. doliatus*, *Pterocaesio capricornis*, and *P. tile*).

**Table 1:** Summary statistics of the genetic distances (K2P) with increasing taxonomic levels

Label	*n*	Taxa	Comparisons	Min dist(%)	Mean dist(%)	Max dist(%)
Within species	643	153	1,268	0.00	0.32	7.12
Within genus	601	35	7,555	0.00	12.05	21.83
Within family	608	17	9,203	7.21	18.28	26.15

**Figure 4:**
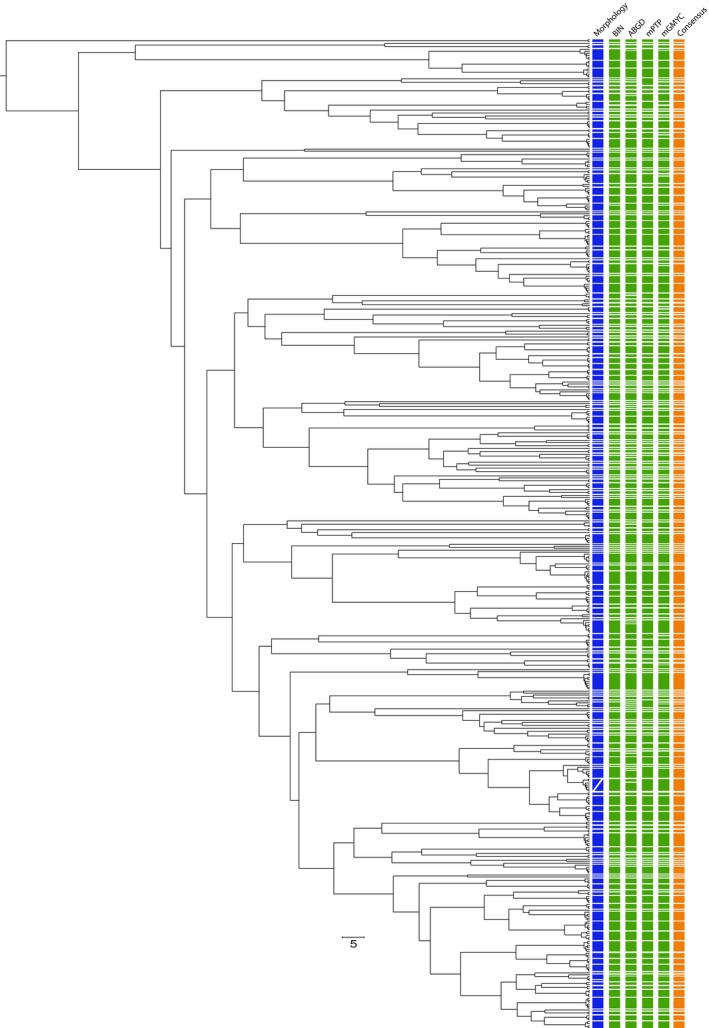
Bayesian Chronogram for the 676 DNA barcodes obtained based on a 1.2% of genetic divergence per Million years including species delimitation scheme based on morphological characters (blue), DNA‐based species delimitation schemes derived from RESL, ABGD, mPTP and mGMYC (green), and the consensus delimitation scheme (orange)

The OTU delimitation analyses yielded varying numbers of OTUs depending on the algorithm used (Figure 4 and Table S3). Numbers of delimited OTUs were 206 for RESL, 217 for ABGD, 160 for mPTP, and 216 for mGMYC (Figure 4 and Table S3). The consensus delimitation scheme yielded 208 OTUs for 202 nominal species, highlighting several conflicts between OTUs and species delimitation (Figure 4 and Table S3). A total of eight species with multiple OTUs were detected with K2P genetic distances ranging from 1.72 in *Priacanthus hamrur* to 3.86 in *Lethrinus ornatus* (Table 2). Three OTUs, however, were shared by more than one species (OTU192 including *S. analiculatus* and *S. fuscescens*, OTU92 including *L. decussatus* and *L. semicinctus*, and OTU196 including *Siganus. guttatus*, *S. lineatus*, and *S. vermiculatus*) for a total of seven species displaying mixed genealogies (Figure S1 and Table S2).

**Table 2:** Summary statistics of the 16 OTUs among the 8 species with more than a single OTU including their BIN, maximum intraspecific distance and distance to the nearest neighbor

	Dist. Max. intra.	Dist. Near. Neigh.
*Lethrinus ornatus*	3.86	7.45
OTU81 (BIN:AAK9232)	0.8	3.86
OTU82 (BIN:AAE7226)	—	3.86
*Lutjanus decussatus*	3.34	0
OTU91 (BIN:AAF0336)	0	3.34
OTU92 (BIN:ADF5202)	0.15	0
*Nemipterus furcosus*	2.36	18.47
OTU117 (BIN:ACE4155)	0.46	2.36
OTU118 (BIN:ADF2810)	0.46	2.36
*Paracaesio xanthura*	2.21	10.27
OTU131 (BIN:AAI6821)	—	2.21
OTU132 (BIN:ABZ2393)	0.33	2.21
*Plectorhinchus lineatus*	1.87	4.81
OTU152 (BIN:AAF2980)	0.31	1.87
OTU153 (BIN:AAF2980)	—	1.87
*Priacanthus hamrur*	1.72	15.05
OTU158 (BIN:AAB1643)	0.78	1.72
OTU159 (BIN:AAB1643)	—	1.72
*Siganus vermiculatus*	3.64	0.31
OTU197 (BIN:ABB2342)	—	0.31
OTU202 (BIN:AAJ6703)	—	3.64
*Siganus caniculatus*	7.12	0
OTU193 (BIN:AAB8846)	0	0
OTU194 (BIN:ACR6962)	—	7.12

## 4 DISCUSSION

The usefulness of DNA barcoding to identify fish species is widely accepted with nearly 90% of the species analyzed having their boundaries aptly captured by DNA barcodes in previous studies (April, Mayden, Hanner, & Bernatchez, 2011; Hubert et al., 2008; Knebelsberger, Dunz, Neumann, & Geiger, 2015; Shen et al., 2019; Weigt et al., 2012). In the present study, only six cases of shared haplotypes between species were detected. Hence, 97% of the species analyzed (196 out of 202) were accurately delimitated by their DNA barcodes. Only 4% of the species with more than one individual sampled showed maximum intraspecific K2P genetic distances lower than the distance to the nearest neighbor (6 out of 153); however, these species were characterized by collections of private haplotypes indicating that they can readily by identified through DNA barcodes (Collins & Cruickshank, 2014). This trend was confirmed by the ratio between the maximum intraspecific and the nearest neighbor genetic distances, the latter being 30‐fold higher on average than the former. This ratio varies substantially between fish studies (7–30) largely depending on the spatial scope and taxonomic coverage (April et al., 2011; Hubert et al., 2017; Pereira, Pazian, Hanner, Foresti, & Oliveira, 2011). The ratio recovered for this study is high indicating that most species consist of tightly defined clusters of DNA barcodes. It may decrease with increasing spatial and taxonomic scale given the high levels of intraspecific genetic distances that have been previously reported for Indo‐Pacific reef fishes at the regional scale (Hubert et al., 2012, 2017; Steinke et al., 2009; Winterbottom et al., 2014). This trend for Indo‐Pacific reef fishes is due to high levels of cryptic diversity especially when species with distribution ranges spanning across the Indian and Pacific ocean are included (Hubert et al., 2017; Zemlak, Ward, Connell, Holmes, & Hebert, 2009). This study confirms, however, that at the local scale, DNA barcoding—less prone to inflated intraspecific genetic distances due to taxonomic uncertainties and cryptic diversity—can be successfully applied to automated identifications of fishes. We detected several cases of cryptic diversity in 4% of the species under scrutiny (16 OTUs delineated within 8 species)' by 'We detected several cases of cryptic diversity (16 OTUs delineated within 8 species), representing 4% of the species under scrutiny. This rate is lower than in previous assessments at regional scale in the Indian and Pacific oceans where 10% of species contained cryptic and highly divergent lineages (Hubert et al., 2012, 2017). Several cases of shared haplotypes were also detected within *Lutjanus* and particularly *Siganus* species. Those potentially result from introgressive hybridization and/or overlapping morphological characters. Several cases were expected as mitochondrial introgression was previously reported for *Siganus* based on a comprehensive assessment of genetic diversity at mitochondrial and nuclear markers (Kuriiwa, Hanzawa, Yoshino, Kimura, & Nishida, 2007; Ravago‐Gotanco, Cruz, Josefa Pante, & Borsa, 2018). In such cases that constitute a limit where sister‐species co‐occur and hybridize (Ravago‐Gotanco et al., 2018), mitochondrial markers will inflate the number of false positives (i.e*.* considering heterospecifics as conspecifics). The amount of false positives detected does not exceed 4% of the specimens analyzed suggesting that, if cautiously interpreting the individual assignment for those species, automated identification can be performed (Collins & Cruickshank, 2014).

This study highlights that artisanal fisheries in the CT harvest a substantial amount of species that originate from a diversity of shore habitats including coral reefs (Serranidae, Holocentridae, Scaridae, Pomacentridae, and Chaetodontidae), seaweed beds (Siganidae, Mullidae, and Labridae), and open waters (Carangidae). The retailed coral reef families account for most of the species richness, for example, the Serranidae (groupers), Acanthuridae (Surgeon fish), and Holocentridae (Soldier fish) that rank first, fifth, and seventh in species number collected, respectively. On the other hand, species‐rich coral reef families such as Pomacentridae and Chaetodontidae were rarely encountered at the fish stalls and only a few species were collected for each family. Our result suggests a market trend toward species from the families Serranidae (*Epinephelus* and *Cephalopholis*) and Lutjanidae (*Lutjanus*) as both rank first and second in terms of number of species landed, respectively. This trend is of particular concern for the sustainability of the CT fisheries as groupers (Serranidae) have already been identified as severely threatened by overfishing (Sadovy de Mitcheson et al., 2013). Both families (with 138 Serranidae and 59 Lutjanidae species) are also not among the most species‐rich in the Indonesian archipelago (Froese & Pauly, 2014), which amplifies the pressure through increased fisheries.

This study confirms the potential of DNA barcoding for the automated identification of Indo‐Pacific shore fishes (Delrieu‐Trottin et al.,^2019^; Durand et al., 2017; Hubert et al., 2017, 2015, 2012; Jaafar, Taylor, Mohd Nor, Bruyn, & Carvalho, 2012; Randall & Victor, 2015; Steinke et al., 2009; Ward, Costa, Holmes, & Steinke, 2008; Winterbottom et al., 2014) and opens new perspectives for the monitoring of CT fisheries. By providing the first reference library available to date for commercial shore fishes of the CT, this study enables the DNA‐based assignment of unknown individuals to known species. In extremely diversified biomes, identification of fish species through morphological characters is a difficult task that relies on a few specialists worldwide. As a consequence of the worldwide loss of taxonomists, Indo‐Pacific shore fish taxonomy has largely became a black box for many groups (Hubert & Hanner, 2015). This development might have also led to the biomass‐based approach to the study of shore fisheries trends in the Indo‐Pacific (Cinner et al., 2013, 2016; Maire et al., 2016). While biomass is certainly a useful proxy for determining fisheries trends and their impact on ecosystems dynamics, it has its limits, particularly in the context of extreme species richness as observed in the CT. Biomass and species richness are not correlated for the Indo‐Pacific as a whole. Biomass peaks at peripheral areas of the CT while species richness peaks for intermediate levels of biomass, a trend that has been suggested based on mathematical models (Mouillot & Mouquet, 2006). This trend shows that ecological dynamics for most species‐rich ecosystems are complex and likely rely on a large array of drivers including their biogeographical history, community assembly dynamics and persistence through time (Gaboriau et al., 2018; Gaither & Rocha, 2013; Hubert et al., 2012; Pellissier et al., 2014). Thus, monitoring ecosystem recovery by measuring biomass might be misleading given that species richness is not directly linked to it but rather associated with a few productive species. In this context, this DNA barcodes reference library opens new possibilities for the monitoring of CT artisanal fisheries by enabling the assessment of their dynamics at the species level and further allows environmental DNA (eDNA) approaches in the CT. Alternative molecular markers have been used for eDNA purposes such as 12S and 16S (Miya et al., 2015), Cytochrome b (Ficetola, Miaud, Pompanon, & Taberlet, 2008), and COI (Hajibabaei, Shokralla, Zhou, Singer, & Baird, 2011) during the last decade. We advocate here for the use of standardized procedures in DNA barcoding along others (Andújar, Arribas, Yu, Vogler, & Emerson, 2018) and recommend the use of the COI gene for eDNA considering the extensive coverage of the COI libraries available (BOLD), the appropriate rate of substitution of this protein‐coding gene for species diagnostic and the development of new sequencing protocols that avoid PCR‐based enrichment (Mariac et al., 2018).

## 5 CONCLUSIONS

This study provides the first DNA barcode reference library of the CT shore fishes targeted by artisanal fisheries. It contains 202 species including collection and sequence data for 696 specimens. Species boundaries were aptly captured by DNA barcodes in 96% of the species examined, which demonstrates the effectiveness of DNA barcodes for further automated identification of unknown specimens. This library opens new perspectives for monitoring of artisanal fisheries in the CT and enables species level surveys. Considering the extreme species richness of the CT marine ecosystems and the difficulties to accurately and routinely identify species, this library allows further detailed studies in fisheries and coastal management.

## CONFLICT OF INTEREST

None declared.

## AUTHORS' CONTRIBUTIONS

GL and NH designed the study. GL, JP, and FR conducted the market survey. GL, JP, HD, DS, and NH conducted the genetic analyzes. GL, JP, FR, EDT, and NH performed the morphological identifications. GL, EDT, and NH analyzed the data. EDT and FB curated the DNA barcode records in BOLD. NH and GL wrote the initial draft of the manuscript, and all authors commented and approved the final version of the manuscript.

## Data Availability

All collecting and sequence data are available on the Barcode of Life Datasystem (BOLD) in the project “Barcoding Indonesian Fishes ‐ DNA Barcoding of the marine fishes landed at Ambon harbour” and are also available on Genbank (see Table S1).
