# Supplementary material for: Assessing species diversity of Coral Triangle artisanal fisheries: A DNA barcode reference library for the shore fishes retailed at Ambon harbor (Indonesia)
Source: Ecol Evol. 2020 Mar 6;10(7):3356–66. doi: 10.1002/ece3.6128 (PMC7141007; doi:10.1002/ece3.6128)
Supplement: Supplementary file 1 [file ECE3-10-3356-s001.pdf]

BOLD TaxonID Tree

Title	:	Tree Result - DBMFA
Date	:	20-May-2019
Data Type	:	Nucleotide
Distance Model	:	Kimura 2 Parameter
Marker	:	COI-5P
Colourization	:	[blue]=Stop Codons [red]=Contamination or misidentification

Label	:	Sample ID
Label	:	Process ID
Label	:	Taxon
Label	:	Barcode Cluster (BIN)

Sequence Count	:	696
Species count	:	202
Genus count	:	73
Family count	:	24
Unidentified	:	4

BIN Count	:	205

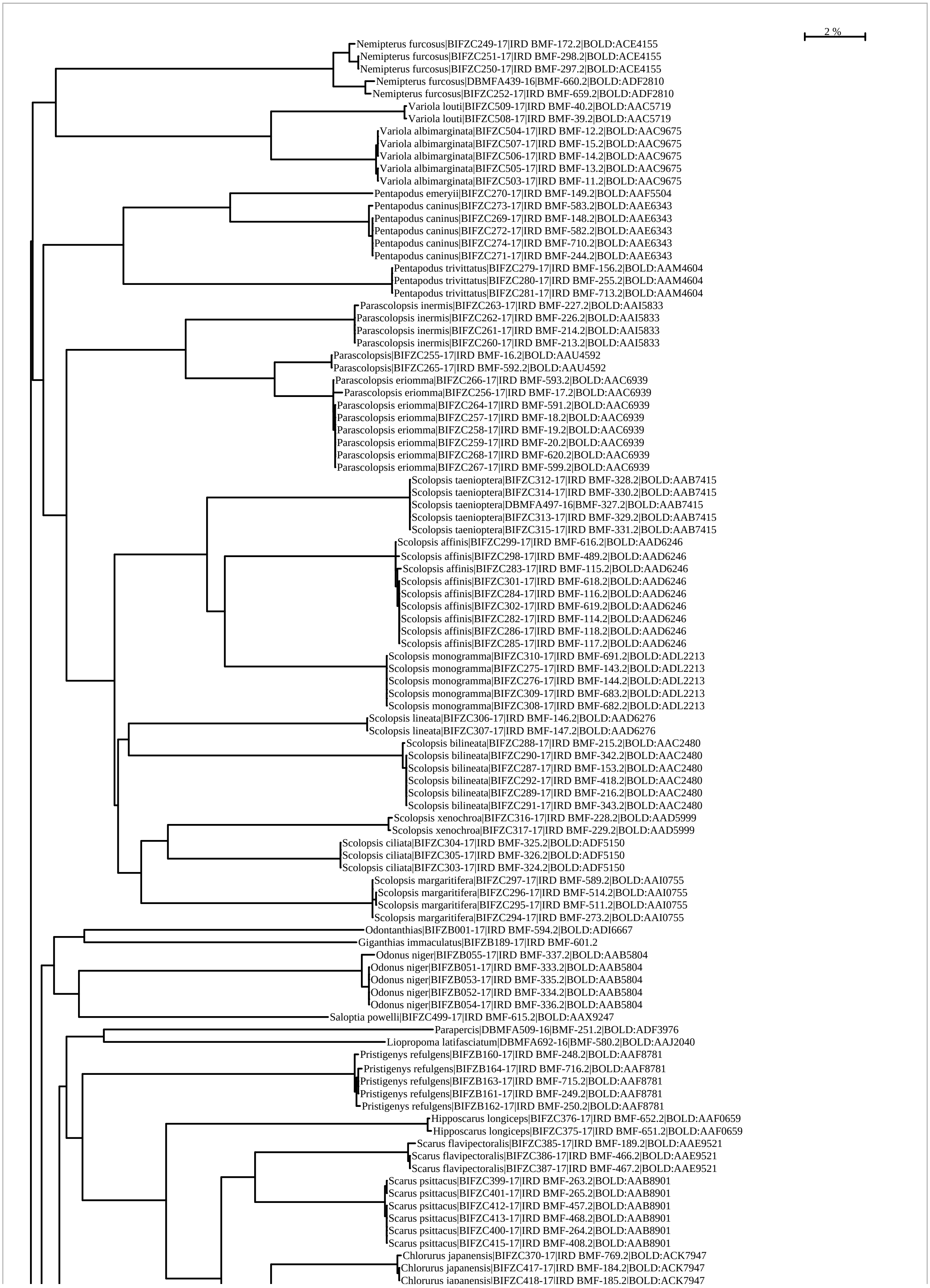
2 %
Nemipterus furcosus|BIFZC249-17|IRD BMF-172.2|BOLD:ACE4155
Nemipterus furcosus|BIFZC251-17|IRD BMF-298.2|BOLD:ACE4155
Nemipterus furcosus|BIFZC250-17|IRD BMF-297.2|BOLD:ACE4155
Nemipterus furcosus|DBMFA439-16|BMF-660.2|BOLD:ADF2810
Nemipterus furcosus|BIFZC252-17|IRD BMF-659.2|BOLD:ADF2810
Variola louti|BIFZC509-17|IRD BMF-40.2|BOLD:AAC5719
Variola louti|BIFZC508-17|IRD BMF-39.2|BOLD:AAC5719
Variola albimarginata|BIFZC504-17|IRD BMF-12.2|BOLD:AAC9675
Variola albimarginata|BIFZC507-17|IRD BMF-15.2|BOLD:AAC9675
Variola albimarginata|BIFZC506-17|IRD BMF-14.2|BOLD:AAC9675
Variola albimarginata|BIFZC505-17|IRD BMF-13.2|BOLD:AAC9675
Variola albimarginata|BIFZC503-17|IRD BMF-11.2|BOLD:AAC9675
Pentapodus emeryii|BIFZC270-17|IRD BMF-149.2|BOLD:AAF5504
Pentapodus caninus|BIFZC273-17|IRD BMF-583.2|BOLD:AAE6343
Pentapodus caninus|BIFZC269-17|IRD BMF-148.2|BOLD:AAE6343
Pentapodus caninus|BIFZC272-17|IRD BMF-582.2|BOLD:AAE6343
Pentapodus caninus|BIFZC274-17|IRD BMF-710.2|BOLD:AAE6343
Pentapodus caninus|BIFZC271-17|IRD BMF-244.2|BOLD:AAE6343
Pentapodus trivittatus|BIFZC279-17|IRD BMF-156.2|BOLD:AAM4604
Pentapodus trivittatus|BIFZC280-17|IRD BMF-255.2|BOLD:AAM4604
Pentapodus trivittatus|BIFZC281-17|IRD BMF-713.2|BOLD:AAM4604
Parascolopsis inermis|BIFZC263-17|IRD BMF-227.2|BOLD:AAI5833
Parascolopsis inermis|BIFZC262-17|IRD BMF-226.2|BOLD:AAI5833
Parascolopsis inermis|BIFZC261-17|IRD BMF-214.2|BOLD:AAI5833
Parascolopsis inermis|BIFZC260-17|IRD BMF-213.2|BOLD:AAI5833
Parascolopsis|BIFZC255-17|IRD BMF-16.2|BOLD:AAU4592
Parascolopsis|BIFZC265-17|IRD BMF-592.2|BOLD:AAU4592
Parascolopsis eriomma|BIFZC266-17|IRD BMF-593.2|BOLD:AAC6939
Parascolopsis eriomma|BIFZC256-17|IRD BMF-17.2|BOLD:AAC6939
Parascolopsis eriomma|BIFZC264-17|IRD BMF-591.2|BOLD:AAC6939
Parascolopsis eriomma|BIFZC257-17|IRD BMF-18.2|BOLD:AAC6939
Parascolopsis eriomma|BIFZC258-17|IRD BMF-19.2|BOLD:AAC6939
Parascolopsis eriomma|BIFZC259-17|IRD BMF-20.2|BOLD:AAC6939
Parascolopsis eriomma|BIFZC268-17|IRD BMF-620.2|BOLD:AAC6939
Parascolopsis eriomma|BIFZC267-17|IRD BMF-599.2|BOLD:AAC6939
Scolopsis taenioptera|BIFZC312-17|IRD BMF-328.2|BOLD:AAB7415
Scolopsis taenioptera|BIFZC314-17|IRD BMF-330.2|BOLD:AAB7415
Scolopsis taenioptera|DBMFA497-16|BMF-327.2|BOLD:AAB7415
Scolopsis taenioptera|BIFZC313-17|IRD BMF-329.2|BOLD:AAB7415
Scolopsis taenioptera|BIFZC315-17|IRD BMF-331.2|BOLD:AAB7415
Scolopsis affinis|BIFZC299-17|IRD BMF-616.2|BOLD:AAD6246
Scolopsis affinis|BIFZC298-17|IRD BMF-489.2|BOLD:AAD6246
Scolopsis affinis|BIFZC283-17|IRD BMF-115.2|BOLD:AAD6246
Scolopsis affinis|BIFZC301-17|IRD BMF-618.2|BOLD:AAD6246
Scolopsis affinis|BIFZC284-17|IRD BMF-116.2|BOLD:AAD6246
Scolopsis affinis|BIFZC302-17|IRD BMF-619.2|BOLD:AAD6246
Scolopsis affinis|BIFZC282-17|IRD BMF-114.2|BOLD:AAD6246
Scolopsis affinis|BIFZC286-17|IRD BMF-118.2|BOLD:AAD6246
Scolopsis affinis|BIFZC285-17|IRD BMF-117.2|BOLD:AAD6246
Scolopsis monogramma|BIFZC310-17|IRD BMF-691.2|BOLD:ADL2213
Scolopsis monogramma|BIFZC275-17|IRD BMF-143.2|BOLD:ADL2213
Scolopsis monogramma|BIFZC276-17|IRD BMF-144.2|BOLD:ADL2213
Scolopsis monogramma|BIFZC309-17|IRD BMF-683.2|BOLD:ADL2213
Scolopsis monogramma|BIFZC308-17|IRD BMF-682.2|BOLD:ADL2213
Scolopsis lineata|BIFZC306-17|IRD BMF-146.2|BOLD:AAD6276
Scolopsis lineata|BIFZC307-17|IRD BMF-147.2|BOLD:AAD6276
Scolopsis bilineata|BIFZC288-17|IRD BMF-215.2|BOLD:AAC2480
Scolopsis bilineata|BIFZC290-17|IRD BMF-342.2|BOLD:AAC2480
Scolopsis bilineata|BIFZC287-17|IRD BMF-153.2|BOLD:AAC2480
Scolopsis bilineata|BIFZC292-17|IRD BMF-418.2|BOLD:AAC2480
Scolopsis bilineata|BIFZC289-17|IRD BMF-216.2|BOLD:AAC2480
Scolopsis bilineata|BIFZC291-17|IRD BMF-343.2|BOLD:AAC2480
Scolopsis xenochroa|BIFZC316-17|IRD BMF-228.2|BOLD:AAD5999
Scolopsis xenochroa|BIFZC317-17|IRD BMF-229.2|BOLD:AAD5999
Scolopsis ciliata|BIFZC304-17|IRD BMF-325.2|BOLD:ADF5150
Scolopsis ciliata|BIFZC305-17|IRD BMF-326.2|BOLD:ADF5150
Scolopsis ciliata|BIFZC303-17|IRD BMF-324.2|BOLD:ADF5150
Scolopsis margaritifera|BIFZC297-17|IRD BMF-589.2|BOLD:AAI0755
Scolopsis margaritifera|BIFZC296-17|IRD BMF-514.2|BOLD:AAI0755
Scolopsis margaritifera|BIFZC295-17|IRD BMF-511.2|BOLD:AAI0755
Scolopsis margaritifera|BIFZC294-17|IRD BMF-273.2|BOLD:AAI0755
Odontanthias|BIFZB001-17|IRD BMF-594.2|BOLD:ADI6667
Giganthias immaculatus|BIFZB189-17|IRD BMF-601.2
Odonus niger|BIFZB055-17|IRD BMF-337.2|BOLD:AAB5804
Odonus niger|BIFZB051-17|IRD BMF-333.2|BOLD:AAB5804
Odonus niger|BIFZB053-17|IRD BMF-335.2|BOLD:AAB5804
Odonus niger|BIFZB052-17|IRD BMF-334.2|BOLD:AAB5804
Odonus niger|BIFZB054-17|IRD BMF-336.2|BOLD:AAB5804
Saloptia powelli|BIFZC499-17|IRD BMF-615.2|BOLD:AAX9247
Parapercis|DBMFA509-16|BMF-251.2|BOLD:ADF3976
Liopropoma latifasciatum|DBMFA692-16|BMF-580.2|BOLD:AAJ2040
Pristigenys refulgens|BIFZB160-17|IRD BMF-248.2|BOLD:AAF8781
Pristigenys refulgens|BIFZB164-17|IRD BMF-716.2|BOLD:AAF8781
Pristigenys refulgens|BIFZB163-17|IRD BMF-715.2|BOLD:AAF8781
Pristigenys refulgens|BIFZB161-17|IRD BMF-249.2|BOLD:AAF8781
Pristigenys refulgens|BIFZB162-17|IRD BMF-250.2|BOLD:AAF8781
Hipposcarus longiceps|BIFZC376-17|IRD BMF-652.2|BOLD:AAF0659
Hipposcarus longiceps|BIFZC375-17|IRD BMF-651.2|BOLD:AAF0659
Scarus flavipectoralis|BIFZC385-17|IRD BMF-189.2|BOLD:AAE9521
Scarus flavipectoralis|BIFZC386-17|IRD BMF-466.2|BOLD:AAE9521
Scarus flavipectoralis|BIFZC387-17|IRD BMF-467.2|BOLD:AAE9521
Scarus psittacus|BIFZC399-17|IRD BMF-263.2|BOLD:AAB8901
Scarus psittacus|BIFZC401-17|IRD BMF-265.2|BOLD:AAB8901
Scarus psittacus|BIFZC412-17|IRD BMF-457.2|BOLD:AAB8901
Scarus psittacus|BIFZC413-17|IRD BMF-468.2|BOLD:AAB8901
Scarus psittacus|BIFZC400-17|IRD BMF-264.2|BOLD:AAB8901
Scarus psittacus|BIFZC415-17|IRD BMF-408.2|BOLD:AAB8901
Chlorurus japanensis|BIFZC370-17|IRD BMF-769.2|BOLD:ACK7947
Chlorurus japanensis|BIFZC417-17|IRD BMF-184.2|BOLD:ACK7947
Chlorurus japanensis|BIFZC418-17|IRD BMF-185.2|BOLD:ACK7947

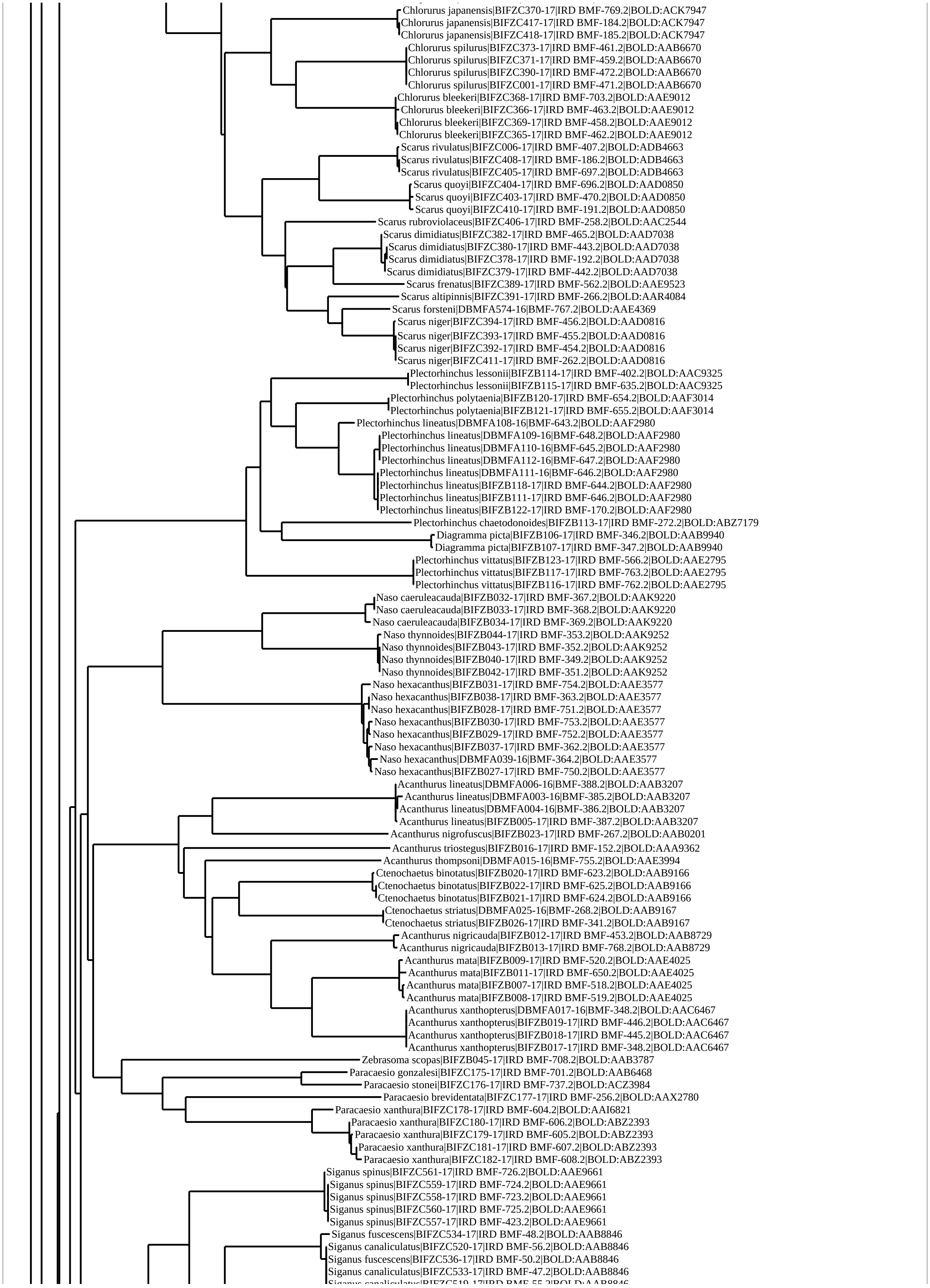
Chlorurus japanensis|BIFZC370-17|IRD BMF-769.2|BOLD:ACK7947
Chlorurus japanensis|BIFZC417-17|IRD BMF-184.2|BOLD:ACK7947
Chlorurus japanensis|BIFZC418-17|IRD BMF-185.2|BOLD:ACK7947
Chlorurus spilurus|BIFZC373-17|IRD BMF-461.2|BOLD:AAB6670
Chlorurus spilurus|BIFZC371-17|IRD BMF-459.2|BOLD:AAB6670
Chlorurus spilurus|BIFZC390-17|IRD BMF-472.2|BOLD:AAB6670
Chlorurus spilurus|BIFZC001-17|IRD BMF-471.2|BOLD:AAB6670
Chlorurus bleekeri|BIFZC368-17|IRD BMF-703.2|BOLD:AAE9012
Chlorurus bleekeri|BIFZC366-17|IRD BMF-463.2|BOLD:AAE9012
Chlorurus bleekeri|BIFZC369-17|IRD BMF-458.2|BOLD:AAE9012
Chlorurus bleekeri|BIFZC365-17|IRD BMF-462.2|BOLD:AAE9012
Scarus rivulatus|BIFZC006-17|IRD BMF-407.2|BOLD:ADB4663
Scarus rivulatus|BIFZC408-17|IRD BMF-186.2|BOLD:ADB4663
Scarus rivulatus|BIFZC405-17|IRD BMF-697.2|BOLD:ADB4663
Scarus quoyi|BIFZC404-17|IRD BMF-696.2|BOLD:AAD0850
Scarus quoyi|BIFZC403-17|IRD BMF-470.2|BOLD:AAD0850
Scarus quoyi|BIFZC410-17|IRD BMF-191.2|BOLD:AAD0850
Scarus rubroviolaceus|BIFZC406-17|IRD BMF-258.2|BOLD:AAC2544
Scarus dimidiatus|BIFZC382-17|IRD BMF-465.2|BOLD:AAD7038
Scarus dimidiatus|BIFZC380-17|IRD BMF-443.2|BOLD:AAD7038
Scarus dimidiatus|BIFZC378-17|IRD BMF-192.2|BOLD:AAD7038
Scarus dimidiatus|BIFZC379-17|IRD BMF-442.2|BOLD:AAD7038
Scarus frenatus|BIFZC389-17|IRD BMF-562.2|BOLD:AAE9523
Scarus altipinnis|BIFZC391-17|IRD BMF-266.2|BOLD:AAR4084
Scarus forsteni|DBMFA574-16|BMF-767.2|BOLD:AAE4369
Scarus niger|BIFZC394-17|IRD BMF-456.2|BOLD:AAD0816
Scarus niger|BIFZC393-17|IRD BMF-455.2|BOLD:AAD0816
Scarus niger|BIFZC392-17|IRD BMF-454.2|BOLD:AAD0816
Scarus niger|BIFZC411-17|IRD BMF-262.2|BOLD:AAD0816
Plectorhinchus lessonii|BIFZB114-17|IRD BMF-402.2|BOLD:AAC9325
Plectorhinchus lessonii|BIFZB115-17|IRD BMF-635.2|BOLD:AAC9325
Plectorhinchus polytaenia|BIFZB120-17|IRD BMF-654.2|BOLD:AAF3014
Plectorhinchus polytaenia|BIFZB121-17|IRD BMF-655.2|BOLD:AAF3014
Plectorhinchus lineatus|DBMFA108-16|BMF-643.2|BOLD:AAF2980
Plectorhinchus lineatus|DBMFA109-16|BMF-648.2|BOLD:AAF2980
Plectorhinchus lineatus|DBMFA110-16|BMF-645.2|BOLD:AAF2980
Plectorhinchus lineatus|DBMFA112-16|BMF-647.2|BOLD:AAF2980
Plectorhinchus lineatus|DBMFA111-16|BMF-646.2|BOLD:AAF2980
Plectorhinchus lineatus|BIFZB118-17|IRD BMF-644.2|BOLD:AAF2980
Plectorhinchus lineatus|BIFZB111-17|IRD BMF-646.2|BOLD:AAF2980
Plectorhinchus lineatus|BIFZB122-17|IRD BMF-170.2|BOLD:AAF2980
Plectorhinchus chaetodonoides|BIFZB113-17|IRD BMF-272.2|BOLD:ABZ7179
Diagramma picta|BIFZB106-17|IRD BMF-346.2|BOLD:AAB9940
Diagramma picta|BIFZB107-17|IRD BMF-347.2|BOLD:AAB9940
Plectorhinchus vittatus|BIFZB123-17|IRD BMF-566.2|BOLD:AAE2795
Plectorhinchus vittatus|BIFZB117-17|IRD BMF-763.2|BOLD:AAE2795
Plectorhinchus vittatus|BIFZB116-17|IRD BMF-762.2|BOLD:AAE2795
Naso caeruleacauda|BIFZB032-17|IRD BMF-367.2|BOLD:AAK9220
Naso caeruleacauda|BIFZB033-17|IRD BMF-368.2|BOLD:AAK9220
Naso caeruleacauda|BIFZB034-17|IRD BMF-369.2|BOLD:AAK9220
Naso thynnoides|BIFZB044-17|IRD BMF-353.2|BOLD:AAK9252
Naso thynnoides|BIFZB043-17|IRD BMF-352.2|BOLD:AAK9252
Naso thynnoides|BIFZB040-17|IRD BMF-349.2|BOLD:AAK9252
Naso thynnoides|BIFZB042-17|IRD BMF-351.2|BOLD:AAK9252
Naso hexacanthus|BIFZB031-17|IRD BMF-754.2|BOLD:AAE3577
Naso hexacanthus|BIFZB038-17|IRD BMF-363.2|BOLD:AAE3577
Naso hexacanthus|BIFZB028-17|IRD BMF-751.2|BOLD:AAE3577
Naso hexacanthus|BIFZB030-17|IRD BMF-753.2|BOLD:AAE3577
Naso hexacanthus|BIFZB029-17|IRD BMF-752.2|BOLD:AAE3577
Naso hexacanthus|BIFZB037-17|IRD BMF-362.2|BOLD:AAE3577
Naso hexacanthus|DBMFA039-16|BMF-364.2|BOLD:AAE3577
Naso hexacanthus|BIFZB027-17|IRD BMF-750.2|BOLD:AAE3577
Acanthurus lineatus|DBMFA006-16|BMF-388.2|BOLD:AAB3207
Acanthurus lineatus|DBMFA003-16|BMF-385.2|BOLD:AAB3207
Acanthurus lineatus|DBMFA004-16|BMF-386.2|BOLD:AAB3207
Acanthurus lineatus|BIFZB005-17|IRD BMF-387.2|BOLD:AAB3207
Acanthurus nigrofuscus|BIFZB023-17|IRD BMF-267.2|BOLD:AAB0201
Acanthurus triostegus|BIFZB016-17|IRD BMF-152.2|BOLD:AAA9362
Acanthurus thompsoni|DBMFA015-16|BMF-755.2|BOLD:AAE3994
Ctenochaetus binotatus|BIFZB020-17|IRD BMF-623.2|BOLD:AAB9166
Ctenochaetus binotatus|BIFZB022-17|IRD BMF-625.2|BOLD:AAB9166
Ctenochaetus binotatus|BIFZB021-17|IRD BMF-624.2|BOLD:AAB9166
Ctenochaetus striatus|DBMFA025-16|BMF-268.2|BOLD:AAB9167
Ctenochaetus striatus|BIFZB026-17|IRD BMF-341.2|BOLD:AAB9167
Acanthurus nigricauda|BIFZB012-17|IRD BMF-453.2|BOLD:AAB8729
Acanthurus nigricauda|BIFZB013-17|IRD BMF-768.2|BOLD:AAB8729
Acanthurus mata|BIFZB009-17|IRD BMF-520.2|BOLD:AAE4025
Acanthurus mata|BIFZB011-17|IRD BMF-650.2|BOLD:AAE4025
Acanthurus mata|BIFZB007-17|IRD BMF-518.2|BOLD:AAE4025
Acanthurus mata|BIFZB008-17|IRD BMF-519.2|BOLD:AAE4025
Acanthurus xanthopterus|DBMFA017-16|BMF-348.2|BOLD:AAC6467
Acanthurus xanthopterus|BIFZB019-17|IRD BMF-446.2|BOLD:AAC6467
Acanthurus xanthopterus|BIFZB018-17|IRD BMF-445.2|BOLD:AAC6467
Acanthurus xanthopterus|BIFZB017-17|IRD BMF-348.2|BOLD:AAC6467
Zebrasoma scopas|BIFZB045-17|IRD BMF-708.2|BOLD:AAB3787
Paracaesio gonzalesi|BIFZC175-17|IRD BMF-701.2|BOLD:AAB6468
Paracaesio stonei|BIFZC176-17|IRD BMF-737.2|BOLD:ACZ3984
Paracaesio brevidentata|BIFZC177-17|IRD BMF-256.2|BOLD:AAX2780
Paracaesio xanthura|BIFZC178-17|IRD BMF-604.2|BOLD:AAI6821
Paracaesio xanthura|BIFZC180-17|IRD BMF-606.2|BOLD:ABZ2393
Paracaesio xanthura|BIFZC179-17|IRD BMF-605.2|BOLD:ABZ2393
Paracaesio xanthura|BIFZC181-17|IRD BMF-607.2|BOLD:ABZ2393
Paracaesio xanthura|BIFZC182-17|IRD BMF-608.2|BOLD:ABZ2393
Siganus spinus|BIFZC561-17|IRD BMF-726.2|BOLD:AAE9661
Siganus spinus|BIFZC559-17|IRD BMF-724.2|BOLD:AAE9661
Siganus spinus|BIFZC558-17|IRD BMF-723.2|BOLD:AAE9661
Siganus spinus|BIFZC560-17|IRD BMF-725.2|BOLD:AAE9661
Siganus spinus|BIFZC557-17|IRD BMF-423.2|BOLD:AAE9661
Siganus fuscescens|BIFZC534-17|IRD BMF-48.2|BOLD:AAB8846
Siganus canaliculatus|BIFZC520-17|IRD BMF-56.2|BOLD:AAB8846
Siganus fuscescens|BIFZC536-17|IRD BMF-50.2|BOLD:AAB8846
Siganus canaliculatus|BIFZC533-17|IRD BMF-47.2|BOLD:AAB8846

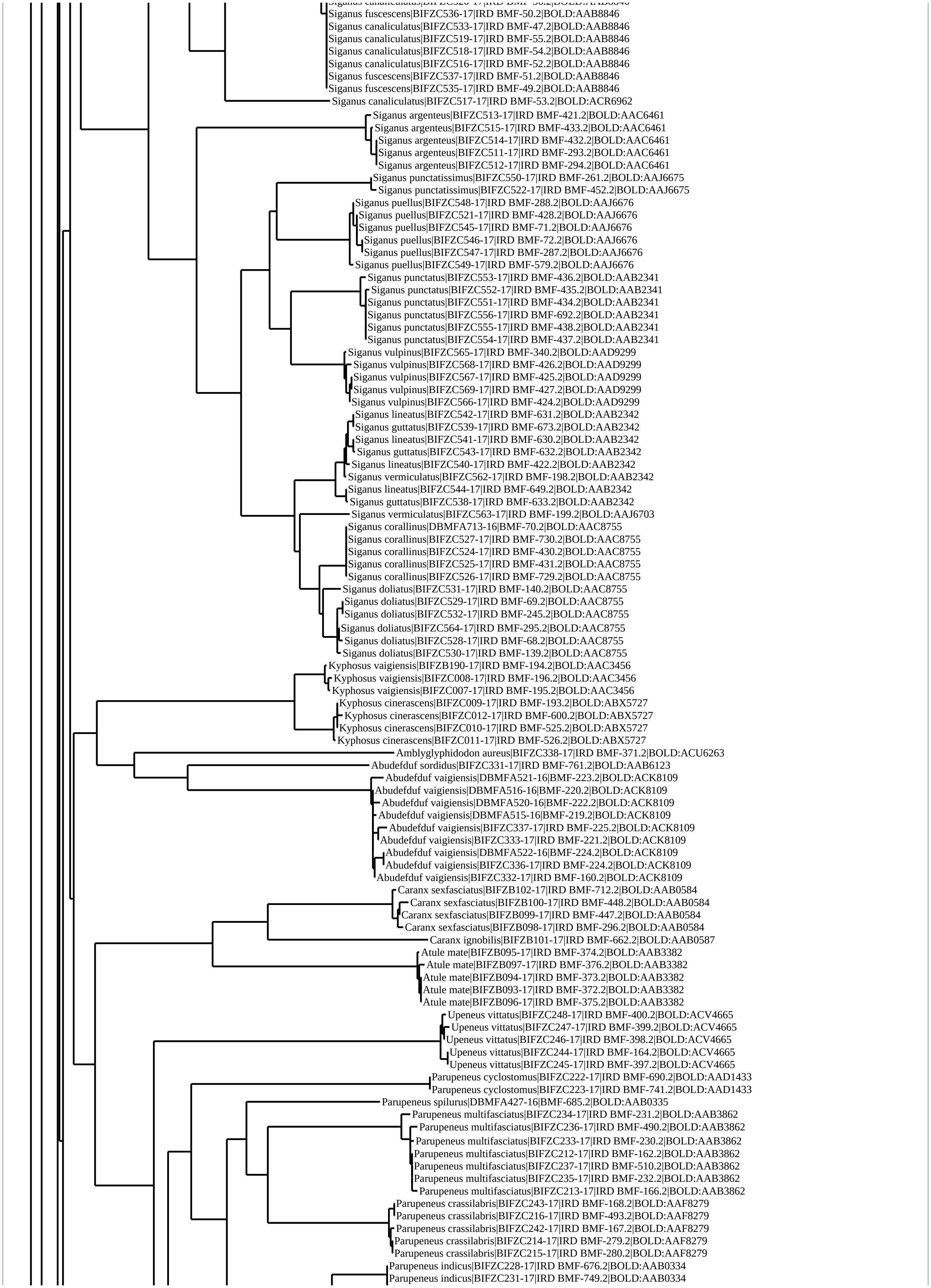
Siganus fuscescens|BIFZC536-17|IRD BMF-50.2|BOLD:AAB8846
Siganus canaliculatus|BIFZC533-17|IRD BMF-47.2|BOLD:AAB8846
Siganus canaliculatus|BIFZC519-17|IRD BMF-55.2|BOLD:AAB8846
Siganus canaliculatus|BIFZC518-17|IRD BMF-54.2|BOLD:AAB8846
Siganus canaliculatus|BIFZC516-17|IRD BMF-52.2|BOLD:AAB8846
Siganus fuscescens|BIFZC537-17|IRD BMF-51.2|BOLD:AAB8846
Siganus fuscescens|BIFZC535-17|IRD BMF-49.2|BOLD:AAB8846
Siganus canaliculatus|BIFZC517-17|IRD BMF-53.2|BOLD:ACR6962
Siganus argenteus|BIFZC513-17|IRD BMF-421.2|BOLD:AAC6461
Siganus argenteus|BIFZC515-17|IRD BMF-433.2|BOLD:AAC6461
Siganus argenteus|BIFZC514-17|IRD BMF-432.2|BOLD:AAC6461
Siganus argenteus|BIFZC511-17|IRD BMF-293.2|BOLD:AAC6461
Siganus argenteus|BIFZC512-17|IRD BMF-294.2|BOLD:AAC6461
Siganus punctatissimus|BIFZC550-17|IRD BMF-261.2|BOLD:AAJ6675
Siganus punctatissimus|BIFZC522-17|IRD BMF-452.2|BOLD:AAJ6675
Siganus puellus|BIFZC548-17|IRD BMF-288.2|BOLD:AAJ6676
Siganus puellus|BIFZC521-17|IRD BMF-428.2|BOLD:AAJ6676
Siganus puellus|BIFZC545-17|IRD BMF-71.2|BOLD:AAJ6676
Siganus puellus|BIFZC546-17|IRD BMF-72.2|BOLD:AAJ6676
Siganus puellus|BIFZC547-17|IRD BMF-287.2|BOLD:AAJ6676
Siganus puellus|BIFZC549-17|IRD BMF-579.2|BOLD:AAJ6676
Siganus punctatus|BIFZC553-17|IRD BMF-436.2|BOLD:AAB2341
Siganus punctatus|BIFZC552-17|IRD BMF-435.2|BOLD:AAB2341
Siganus punctatus|BIFZC551-17|IRD BMF-434.2|BOLD:AAB2341
Siganus punctatus|BIFZC556-17|IRD BMF-692.2|BOLD:AAB2341
Siganus punctatus|BIFZC555-17|IRD BMF-438.2|BOLD:AAB2341
Siganus punctatus|BIFZC554-17|IRD BMF-437.2|BOLD:AAB2341
Siganus vulpinus|BIFZC565-17|IRD BMF-340.2|BOLD:AAD9299
Siganus vulpinus|BIFZC568-17|IRD BMF-426.2|BOLD:AAD9299
Siganus vulpinus|BIFZC567-17|IRD BMF-425.2|BOLD:AAD9299
Siganus vulpinus|BIFZC569-17|IRD BMF-427.2|BOLD:AAD9299
Siganus vulpinus|BIFZC566-17|IRD BMF-424.2|BOLD:AAD9299
Siganus lineatus|BIFZC542-17|IRD BMF-631.2|BOLD:AAB2342
Siganus guttatus|BIFZC539-17|IRD BMF-673.2|BOLD:AAB2342
Siganus lineatus|BIFZC541-17|IRD BMF-630.2|BOLD:AAB2342
Siganus guttatus|BIFZC543-17|IRD BMF-632.2|BOLD:AAB2342
Siganus lineatus|BIFZC540-17|IRD BMF-422.2|BOLD:AAB2342
Siganus vermiculatus|BIFZC562-17|IRD BMF-198.2|BOLD:AAB2342
Siganus lineatus|BIFZC544-17|IRD BMF-649.2|BOLD:AAB2342
Siganus guttatus|BIFZC538-17|IRD BMF-633.2|BOLD:AAB2342
Siganus vermiculatus|BIFZC563-17|IRD BMF-199.2|BOLD:AAJ6703
Siganus corallinus|DBMFA713-16|BMF-70.2|BOLD:AAC8755
Siganus corallinus|BIFZC527-17|IRD BMF-730.2|BOLD:AAC8755
Siganus corallinus|BIFZC524-17|IRD BMF-430.2|BOLD:AAC8755
Siganus corallinus|BIFZC525-17|IRD BMF-431.2|BOLD:AAC8755
Siganus corallinus|BIFZC526-17|IRD BMF-729.2|BOLD:AAC8755
Siganus doliatus|BIFZC531-17|IRD BMF-140.2|BOLD:AAC8755
Siganus doliatus|BIFZC529-17|IRD BMF-69.2|BOLD:AAC8755
Siganus doliatus|BIFZC532-17|IRD BMF-245.2|BOLD:AAC8755
Siganus doliatus|BIFZC564-17|IRD BMF-295.2|BOLD:AAC8755
Siganus doliatus|BIFZC528-17|IRD BMF-68.2|BOLD:AAC8755
Siganus doliatus|BIFZC530-17|IRD BMF-139.2|BOLD:AAC8755
Kyphosus vaigiensis|BIFZB190-17|IRD BMF-194.2|BOLD:AAC3456
Kyphosus vaigiensis|BIFZC008-17|IRD BMF-196.2|BOLD:AAC3456
Kyphosus vaigiensis|BIFZC007-17|IRD BMF-195.2|BOLD:AAC3456
Kyphosus cinerascens|BIFZC009-17|IRD BMF-193.2|BOLD:ABX5727
Kyphosus cinerascens|BIFZC012-17|IRD BMF-600.2|BOLD:ABX5727
Kyphosus cinerascens|BIFZC010-17|IRD BMF-525.2|BOLD:ABX5727
Kyphosus cinerascens|BIFZC011-17|IRD BMF-526.2|BOLD:ABX5727
Amblyglyphidodon aureus|BIFZC338-17|IRD BMF-371.2|BOLD:ACU6263
Abudefduf sordidus|BIFZC331-17|IRD BMF-761.2|BOLD:AAB6123
Abudefduf vaigiensis|DBMFA521-16|BMF-223.2|BOLD:ACK8109
Abudefduf vaigiensis|DBMFA516-16|BMF-220.2|BOLD:ACK8109
Abudefduf vaigiensis|DBMFA520-16|BMF-222.2|BOLD:ACK8109
Abudefduf vaigiensis|DBMFA515-16|BMF-219.2|BOLD:ACK8109
Abudefduf vaigiensis|BIFZC337-17|IRD BMF-225.2|BOLD:ACK8109
Abudefduf vaigiensis|BIFZC333-17|IRD BMF-221.2|BOLD:ACK8109
Abudefduf vaigiensis|DBMFA522-16|BMF-224.2|BOLD:ACK8109
Abudefduf vaigiensis|BIFZC336-17|IRD BMF-224.2|BOLD:ACK8109
Abudefduf vaigiensis|BIFZC332-17|IRD BMF-160.2|BOLD:ACK8109
Caranx sexfasciatus|BIFZB102-17|IRD BMF-712.2|BOLD:AAB0584
Caranx sexfasciatus|BIFZB100-17|IRD BMF-448.2|BOLD:AAB0584
Caranx sexfasciatus|BIFZB099-17|IRD BMF-447.2|BOLD:AAB0584
Caranx sexfasciatus|BIFZB098-17|IRD BMF-296.2|BOLD:AAB0584
Caranx ignobilis|BIFZB101-17|IRD BMF-662.2|BOLD:AAB0587
Atule mate|BIFZB095-17|IRD BMF-374.2|BOLD:AAB3382
Atule mate|BIFZB097-17|IRD BMF-376.2|BOLD:AAB3382
Atule mate|BIFZB094-17|IRD BMF-373.2|BOLD:AAB3382
Atule mate|BIFZB093-17|IRD BMF-372.2|BOLD:AAB3382
Atule mate|BIFZB096-17|IRD BMF-375.2|BOLD:AAB3382
Upeneus vittatus|BIFZC248-17|IRD BMF-400.2|BOLD:ACV4665
Upeneus vittatus|BIFZC247-17|IRD BMF-399.2|BOLD:ACV4665
Upeneus vittatus|BIFZC246-17|IRD BMF-398.2|BOLD:ACV4665
Upeneus vittatus|BIFZC244-17|IRD BMF-164.2|BOLD:ACV4665
Upeneus vittatus|BIFZC245-17|IRD BMF-397.2|BOLD:ACV4665
Parupeneus cyclostomus|BIFZC222-17|IRD BMF-690.2|BOLD:AAD1433
Parupeneus cyclostomus|BIFZC223-17|IRD BMF-741.2|BOLD:AAD1433
Parupeneus spilurus|DBMFA427-16|BMF-685.2|BOLD:AAB0335
Parupeneus multifasciatus|BIFZC234-17|IRD BMF-231.2|BOLD:AAB3862
Parupeneus multifasciatus|BIFZC236-17|IRD BMF-490.2|BOLD:AAB3862
Parupeneus multifasciatus|BIFZC233-17|IRD BMF-230.2|BOLD:AAB3862
Parupeneus multifasciatus|BIFZC212-17|IRD BMF-162.2|BOLD:AAB3862
Parupeneus multifasciatus|BIFZC237-17|IRD BMF-510.2|BOLD:AAB3862
Parupeneus multifasciatus|BIFZC235-17|IRD BMF-232.2|BOLD:AAB3862
Parupeneus multifasciatus|BIFZC213-17|IRD BMF-166.2|BOLD:AAB3862
Parupeneus crassilabris|BIFZC243-17|IRD BMF-168.2|BOLD:AAF8279
Parupeneus crassilabris|BIFZC216-17|IRD BMF-493.2|BOLD:AAF8279
Parupeneus crassilabris|BIFZC242-17|IRD BMF-167.2|BOLD:AAF8279
Parupeneus crassilabris|BIFZC214-17|IRD BMF-279.2|BOLD:AAF8279
Parupeneus crassilabris|BIFZC215-17|IRD BMF-280.2|BOLD:AAF8279
Parupeneus indicus|BIFZC228-17|IRD BMF-676.2|BOLD:AAB0334
Parupeneus indicus|BIFZC231-17|IRD BMF-749.2|BOLD:AAB0334

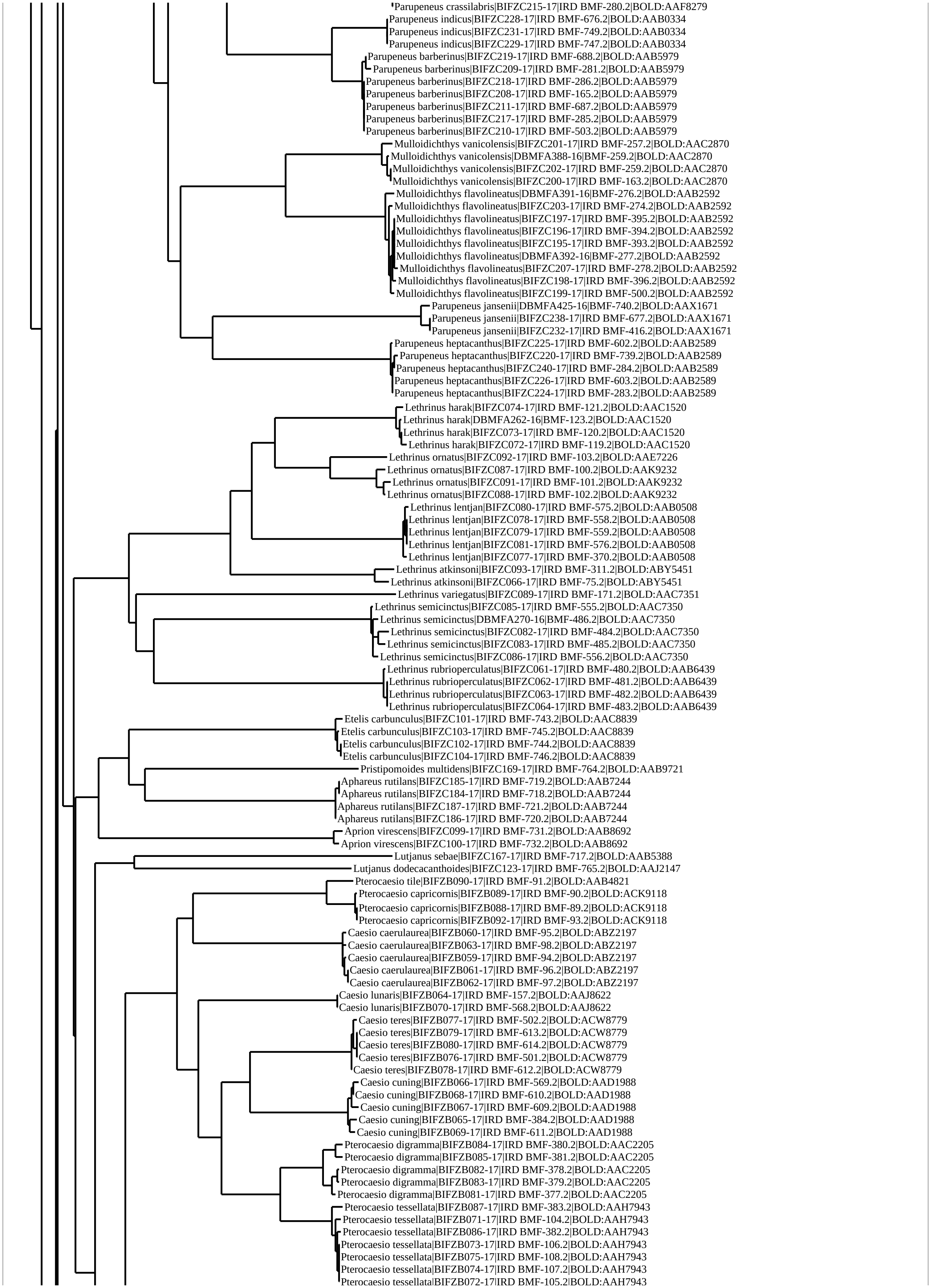
Parupeneus crassilabris|BIFZC215-17|IRD BMF-280.2|BOLD:AAF8279
Parupeneus indicus|BIFZC228-17|IRD BMF-676.2|BOLD:AAB0334
Parupeneus indicus|BIFZC231-17|IRD BMF-749.2|BOLD:AAB0334
Parupeneus indicus|BIFZC229-17|IRD BMF-747.2|BOLD:AAB0334
Parupeneus barberinus|BIFZC219-17|IRD BMF-688.2|BOLD:AAB5979
Parupeneus barberinus|BIFZC209-17|IRD BMF-281.2|BOLD:AAB5979
Parupeneus barberinus|BIFZC218-17|IRD BMF-286.2|BOLD:AAB5979
Parupeneus barberinus|BIFZC208-17|IRD BMF-165.2|BOLD:AAB5979
Parupeneus barberinus|BIFZC211-17|IRD BMF-687.2|BOLD:AAB5979
Parupeneus barberinus|BIFZC217-17|IRD BMF-285.2|BOLD:AAB5979
Parupeneus barberinus|BIFZC210-17|IRD BMF-503.2|BOLD:AAB5979
Mulloidichthys vanicolensis|BIFZC201-17|IRD BMF-257.2|BOLD:AAC2870
Mulloidichthys vanicolensis|DBMFA388-16|BMF-259.2|BOLD:AAC2870
Mulloidichthys vanicolensis|BIFZC202-17|IRD BMF-259.2|BOLD:AAC2870
Mulloidichthys vanicolensis|BIFZC200-17|IRD BMF-163.2|BOLD:AAC2870
Mulloidichthys flavolineatus|DBMFA391-16|BMF-276.2|BOLD:AAB2592
Mulloidichthys flavolineatus|BIFZC203-17|IRD BMF-274.2|BOLD:AAB2592
Mulloidichthys flavolineatus|BIFZC197-17|IRD BMF-395.2|BOLD:AAB2592
Mulloidichthys flavolineatus|BIFZC196-17|IRD BMF-394.2|BOLD:AAB2592
Mulloidichthys flavolineatus|BIFZC195-17|IRD BMF-393.2|BOLD:AAB2592
Mulloidichthys flavolineatus|DBMFA392-16|BMF-277.2|BOLD:AAB2592
Mulloidichthys flavolineatus|BIFZC207-17|IRD BMF-278.2|BOLD:AAB2592
Mulloidichthys flavolineatus|BIFZC198-17|IRD BMF-396.2|BOLD:AAB2592
Mulloidichthys flavolineatus|BIFZC199-17|IRD BMF-500.2|BOLD:AAB2592
Parupeneus jansenii|DBMFA425-16|BMF-740.2|BOLD:AAX1671
Parupeneus jansenii|BIFZC238-17|IRD BMF-677.2|BOLD:AAX1671
Parupeneus jansenii|BIFZC232-17|IRD BMF-416.2|BOLD:AAX1671
Parupeneus heptacanthus|BIFZC225-17|IRD BMF-602.2|BOLD:AAB2589
Parupeneus heptacanthus|BIFZC220-17|IRD BMF-739.2|BOLD:AAB2589
Parupeneus heptacanthus|BIFZC240-17|IRD BMF-284.2|BOLD:AAB2589
Parupeneus heptacanthus|BIFZC226-17|IRD BMF-603.2|BOLD:AAB2589
Parupeneus heptacanthus|BIFZC224-17|IRD BMF-283.2|BOLD:AAB2589
Lethrinus harak|BIFZC074-17|IRD BMF-121.2|BOLD:AAC1520
Lethrinus harak|DBMFA262-16|BMF-123.2|BOLD:AAC1520
Lethrinus harak|BIFZC073-17|IRD BMF-120.2|BOLD:AAC1520
Lethrinus harak|BIFZC072-17|IRD BMF-119.2|BOLD:AAC1520
Lethrinus ornatus|BIFZC092-17|IRD BMF-103.2|BOLD:AAE7226
Lethrinus ornatus|BIFZC087-17|IRD BMF-100.2|BOLD:AAK9232
Lethrinus ornatus|BIFZC091-17|IRD BMF-101.2|BOLD:AAK9232
Lethrinus ornatus|BIFZC088-17|IRD BMF-102.2|BOLD:AAK9232
Lethrinus lentjan|BIFZC080-17|IRD BMF-575.2|BOLD:AAB0508
Lethrinus lentjan|BIFZC078-17|IRD BMF-558.2|BOLD:AAB0508
Lethrinus lentjan|BIFZC079-17|IRD BMF-559.2|BOLD:AAB0508
Lethrinus lentjan|BIFZC081-17|IRD BMF-576.2|BOLD:AAB0508
Lethrinus lentjan|BIFZC077-17|IRD BMF-370.2|BOLD:AAB0508
Lethrinus atkinsoni|BIFZC093-17|IRD BMF-311.2|BOLD:ABY5451
Lethrinus atkinsoni|BIFZC066-17|IRD BMF-75.2|BOLD:ABY5451
Lethrinus variegatus|BIFZC089-17|IRD BMF-171.2|BOLD:AAC7351
Lethrinus semicinctus|BIFZC085-17|IRD BMF-555.2|BOLD:AAC7350
Lethrinus semicinctus|DBMFA270-16|BMF-486.2|BOLD:AAC7350
Lethrinus semicinctus|BIFZC082-17|IRD BMF-484.2|BOLD:AAC7350
Lethrinus semicinctus|BIFZC083-17|IRD BMF-485.2|BOLD:AAC7350
Lethrinus semicinctus|BIFZC086-17|IRD BMF-556.2|BOLD:AAC7350
Lethrinus rubrioperculatus|BIFZC061-17|IRD BMF-480.2|BOLD:AAB6439
Lethrinus rubrioperculatus|BIFZC062-17|IRD BMF-481.2|BOLD:AAB6439
Lethrinus rubrioperculatus|BIFZC063-17|IRD BMF-482.2|BOLD:AAB6439
Lethrinus rubrioperculatus|BIFZC064-17|IRD BMF-483.2|BOLD:AAB6439
Etelis carbunculus|BIFZC101-17|IRD BMF-743.2|BOLD:AAC8839
Etelis carbunculus|BIFZC103-17|IRD BMF-745.2|BOLD:AAC8839
Etelis carbunculus|BIFZC102-17|IRD BMF-744.2|BOLD:AAC8839
Etelis carbunculus|BIFZC104-17|IRD BMF-746.2|BOLD:AAC8839
Pristipomoides multidens|BIFZC169-17|IRD BMF-764.2|BOLD:AAB9721
Aphareus rutilans|BIFZC185-17|IRD BMF-719.2|BOLD:AAB7244
Aphareus rutilans|BIFZC184-17|IRD BMF-718.2|BOLD:AAB7244
Aphareus rutilans|BIFZC187-17|IRD BMF-721.2|BOLD:AAB7244
Aphareus rutilans|BIFZC186-17|IRD BMF-720.2|BOLD:AAB7244
Aprion virescens|BIFZC099-17|IRD BMF-731.2|BOLD:AAB8692
Aprion virescens|BIFZC100-17|IRD BMF-732.2|BOLD:AAB8692
Lutjanus sebae|BIFZC167-17|IRD BMF-717.2|BOLD:AAB5388
Lutjanus dodecacanthoides|BIFZC123-17|IRD BMF-765.2|BOLD:AAJ2147
Pterocaesio tile|BIFZB090-17|IRD BMF-91.2|BOLD:AAB4821
Pterocaesio capricornis|BIFZB089-17|IRD BMF-90.2|BOLD:ACK9118
Pterocaesio capricornis|BIFZB088-17|IRD BMF-89.2|BOLD:ACK9118
Pterocaesio capricornis|BIFZB092-17|IRD BMF-93.2|BOLD:ACK9118
Caesio caerulaurea|BIFZB060-17|IRD BMF-95.2|BOLD:ABZ2197
Caesio caerulaurea|BIFZB063-17|IRD BMF-98.2|BOLD:ABZ2197
Caesio caerulaurea|BIFZB059-17|IRD BMF-94.2|BOLD:ABZ2197
Caesio caerulaurea|BIFZB061-17|IRD BMF-96.2|BOLD:ABZ2197
Caesio caerulaurea|BIFZB062-17|IRD BMF-97.2|BOLD:ABZ2197
Caesio lunaris|BIFZB064-17|IRD BMF-157.2|BOLD:AAJ8622
Caesio lunaris|BIFZB070-17|IRD BMF-568.2|BOLD:AAJ8622
Caesio teres|BIFZB077-17|IRD BMF-502.2|BOLD:ACW8779
Caesio teres|BIFZB079-17|IRD BMF-613.2|BOLD:ACW8779
Caesio teres|BIFZB080-17|IRD BMF-614.2|BOLD:ACW8779
Caesio teres|BIFZB076-17|IRD BMF-501.2|BOLD:ACW8779
Caesio teres|BIFZB078-17|IRD BMF-612.2|BOLD:ACW8779
Caesio cuning|BIFZB066-17|IRD BMF-569.2|BOLD:AAD1988
Caesio cuning|BIFZB068-17|IRD BMF-610.2|BOLD:AAD1988
Caesio cuning|BIFZB067-17|IRD BMF-609.2|BOLD:AAD1988
Caesio cuning|BIFZB065-17|IRD BMF-384.2|BOLD:AAD1988
Caesio cuning|BIFZB069-17|IRD BMF-611.2|BOLD:AAD1988
Pterocaesio digramma|BIFZB084-17|IRD BMF-380.2|BOLD:AAC2205
Pterocaesio digramma|BIFZB085-17|IRD BMF-381.2|BOLD:AAC2205
Pterocaesio digramma|BIFZB082-17|IRD BMF-378.2|BOLD:AAC2205
Pterocaesio digramma|BIFZB083-17|IRD BMF-379.2|BOLD:AAC2205
Pterocaesio digramma|BIFZB081-17|IRD BMF-377.2|BOLD:AAC2205
Pterocaesio tessellata|BIFZB087-17|IRD BMF-383.2|BOLD:AAH7943
Pterocaesio tessellata|BIFZB071-17|IRD BMF-104.2|BOLD:AAH7943
Pterocaesio tessellata|BIFZB086-17|IRD BMF-382.2|BOLD:AAH7943
Pterocaesio tessellata|BIFZB073-17|IRD BMF-106.2|BOLD:AAH7943
Pterocaesio tessellata|BIFZB075-17|IRD BMF-108.2|BOLD:AAH7943
Pterocaesio tessellata|BIFZB074-17|IRD BMF-107.2|BOLD:AAH7943
Pterocaesio tessellata|BIFZB072-17|IRD BMF-105.2|BOLD:AAH7943

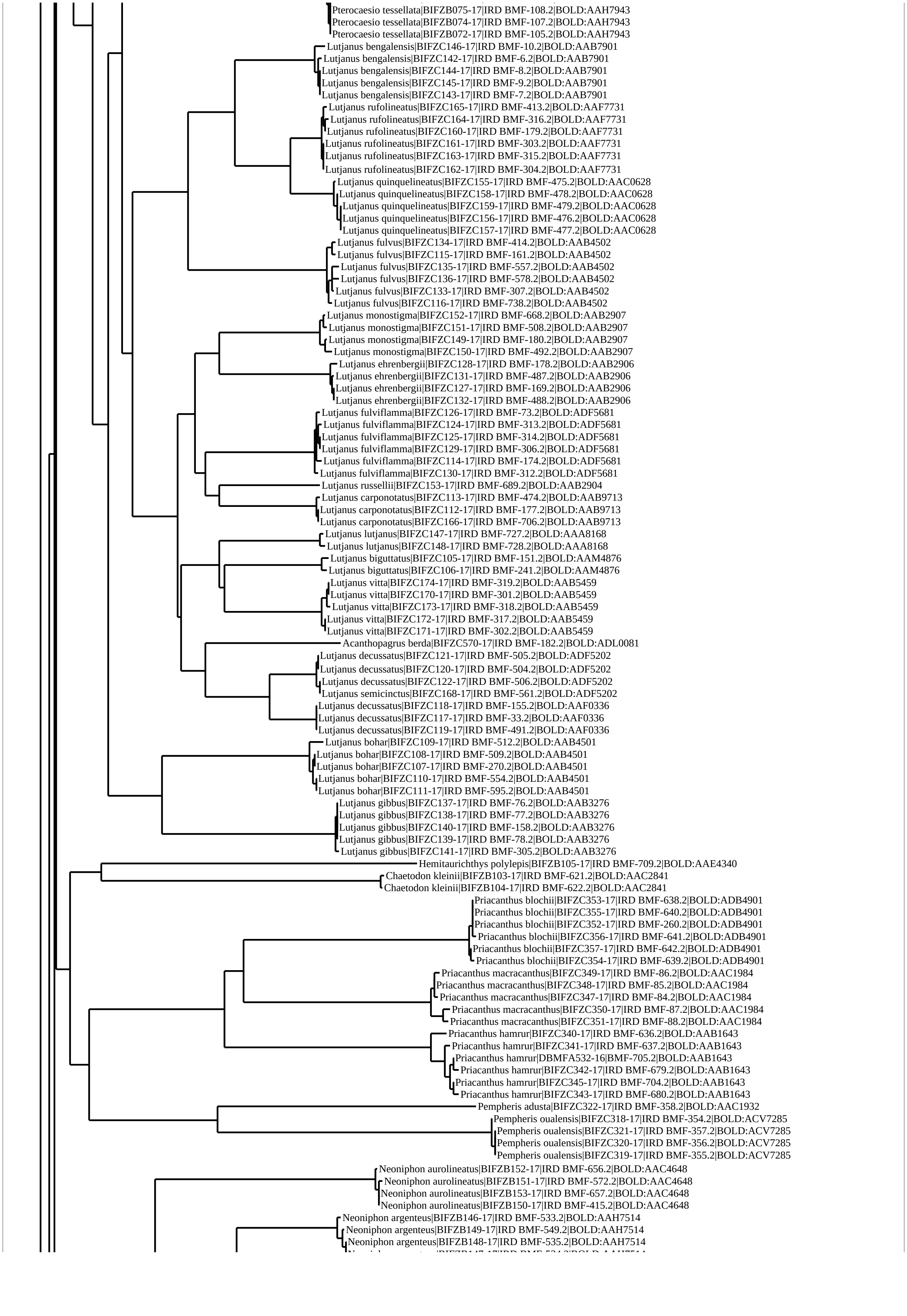
Pterocaesio tessellata|BIFZB075-17|IRD BMF-108.2|BOLD:AAH7943
Pterocaesio tessellata|BIFZB074-17|IRD BMF-107.2|BOLD:AAH7943
Pterocaesio tessellata|BIFZB072-17|IRD BMF-105.2|BOLD:AAH7943
Lutjanus bengalensis|BIFZC146-17|IRD BMF-10.2|BOLD:AAB7901
Lutjanus bengalensis|BIFZC142-17|IRD BMF-6.2|BOLD:AAB7901
Lutjanus bengalensis|BIFZC144-17|IRD BMF-8.2|BOLD:AAB7901
Lutjanus bengalensis|BIFZC145-17|IRD BMF-9.2|BOLD:AAB7901
Lutjanus bengalensis|BIFZC143-17|IRD BMF-7.2|BOLD:AAB7901
Lutjanus rufolineatus|BIFZC165-17|IRD BMF-413.2|BOLD:AAF7731
Lutjanus rufolineatus|BIFZC164-17|IRD BMF-316.2|BOLD:AAF7731
Lutjanus rufolineatus|BIFZC160-17|IRD BMF-179.2|BOLD:AAF7731
Lutjanus rufolineatus|BIFZC161-17|IRD BMF-303.2|BOLD:AAF7731
Lutjanus rufolineatus|BIFZC163-17|IRD BMF-315.2|BOLD:AAF7731
Lutjanus rufolineatus|BIFZC162-17|IRD BMF-304.2|BOLD:AAF7731
Lutjanus quinquelineatus|BIFZC155-17|IRD BMF-475.2|BOLD:AAC0628
Lutjanus quinquelineatus|BIFZC158-17|IRD BMF-478.2|BOLD:AAC0628
Lutjanus quinquelineatus|BIFZC159-17|IRD BMF-479.2|BOLD:AAC0628
Lutjanus quinquelineatus|BIFZC156-17|IRD BMF-476.2|BOLD:AAC0628
Lutjanus quinquelineatus|BIFZC157-17|IRD BMF-477.2|BOLD:AAC0628
Lutjanus fulvus|BIFZC134-17|IRD BMF-414.2|BOLD:AAB4502
Lutjanus fulvus|BIFZC115-17|IRD BMF-161.2|BOLD:AAB4502
Lutjanus fulvus|BIFZC135-17|IRD BMF-557.2|BOLD:AAB4502
Lutjanus fulvus|BIFZC136-17|IRD BMF-578.2|BOLD:AAB4502
Lutjanus fulvus|BIFZC133-17|IRD BMF-307.2|BOLD:AAB4502
Lutjanus fulvus|BIFZC116-17|IRD BMF-738.2|BOLD:AAB4502
Lutjanus monostigma|BIFZC152-17|IRD BMF-668.2|BOLD:AAB2907
Lutjanus monostigma|BIFZC151-17|IRD BMF-508.2|BOLD:AAB2907
Lutjanus monostigma|BIFZC149-17|IRD BMF-180.2|BOLD:AAB2907
Lutjanus monostigma|BIFZC150-17|IRD BMF-492.2|BOLD:AAB2907
Lutjanus ehrenbergii|BIFZC128-17|IRD BMF-178.2|BOLD:AAB2906
Lutjanus ehrenbergii|BIFZC131-17|IRD BMF-487.2|BOLD:AAB2906
Lutjanus ehrenbergii|BIFZC127-17|IRD BMF-169.2|BOLD:AAB2906
Lutjanus ehrenbergii|BIFZC132-17|IRD BMF-488.2|BOLD:AAB2906
Lutjanus fulviflamma|BIFZC126-17|IRD BMF-73.2|BOLD:ADF5681
Lutjanus fulviflamma|BIFZC124-17|IRD BMF-313.2|BOLD:ADF5681
Lutjanus fulviflamma|BIFZC125-17|IRD BMF-314.2|BOLD:ADF5681
Lutjanus fulviflamma|BIFZC129-17|IRD BMF-306.2|BOLD:ADF5681
Lutjanus fulviflamma|BIFZC114-17|IRD BMF-174.2|BOLD:ADF5681
Lutjanus fulviflamma|BIFZC130-17|IRD BMF-312.2|BOLD:ADF5681
Lutjanus russellii|BIFZC153-17|IRD BMF-689.2|BOLD:AAB2904
Lutjanus carponotatus|BIFZC113-17|IRD BMF-474.2|BOLD:AAB9713
Lutjanus carponotatus|BIFZC112-17|IRD BMF-177.2|BOLD:AAB9713
Lutjanus carponotatus|BIFZC166-17|IRD BMF-706.2|BOLD:AAB9713
Lutjanus lutjanus|BIFZC147-17|IRD BMF-727.2|BOLD:AAA8168
Lutjanus lutjanus|BIFZC148-17|IRD BMF-728.2|BOLD:AAA8168
Lutjanus biguttatus|BIFZC105-17|IRD BMF-151.2|BOLD:AAM4876
Lutjanus biguttatus|BIFZC106-17|IRD BMF-241.2|BOLD:AAM4876
Lutjanus vitta|BIFZC174-17|IRD BMF-319.2|BOLD:AAB5459
Lutjanus vitta|BIFZC170-17|IRD BMF-301.2|BOLD:AAB5459
Lutjanus vitta|BIFZC173-17|IRD BMF-318.2|BOLD:AAB5459
Lutjanus vitta|BIFZC172-17|IRD BMF-317.2|BOLD:AAB5459
Lutjanus vitta|BIFZC171-17|IRD BMF-302.2|BOLD:AAB5459
Acanthopagrus berda|BIFZC570-17|IRD BMF-182.2|BOLD:ADL0081
Lutjanus decussatus|BIFZC121-17|IRD BMF-505.2|BOLD:ADF5202
Lutjanus decussatus|BIFZC120-17|IRD BMF-504.2|BOLD:ADF5202
Lutjanus decussatus|BIFZC122-17|IRD BMF-506.2|BOLD:ADF5202
Lutjanus semicinctus|BIFZC168-17|IRD BMF-561.2|BOLD:ADF5202
Lutjanus decussatus|BIFZC118-17|IRD BMF-155.2|BOLD:AAF0336
Lutjanus decussatus|BIFZC117-17|IRD BMF-33.2|BOLD:AAF0336
Lutjanus decussatus|BIFZC119-17|IRD BMF-491.2|BOLD:AAF0336
Lutjanus bohar|BIFZC109-17|IRD BMF-512.2|BOLD:AAB4501
Lutjanus bohar|BIFZC108-17|IRD BMF-509.2|BOLD:AAB4501
Lutjanus bohar|BIFZC107-17|IRD BMF-270.2|BOLD:AAB4501
Lutjanus bohar|BIFZC110-17|IRD BMF-554.2|BOLD:AAB4501
Lutjanus bohar|BIFZC111-17|IRD BMF-595.2|BOLD:AAB4501
Lutjanus gibbus|BIFZC137-17|IRD BMF-76.2|BOLD:AAB3276
Lutjanus gibbus|BIFZC138-17|IRD BMF-77.2|BOLD:AAB3276
Lutjanus gibbus|BIFZC140-17|IRD BMF-158.2|BOLD:AAB3276
Lutjanus gibbus|BIFZC139-17|IRD BMF-78.2|BOLD:AAB3276
Lutjanus gibbus|BIFZC141-17|IRD BMF-305.2|BOLD:AAB3276
Hemitaurichthys polylepis|BIFZB105-17|IRD BMF-709.2|BOLD:AAE4340
Chaetodon kleinii|BIFZB103-17|IRD BMF-621.2|BOLD:AAC2841
Chaetodon kleinii|BIFZB104-17|IRD BMF-622.2|BOLD:AAC2841
Priacanthus blochii|BIFZC353-17|IRD BMF-638.2|BOLD:ADB4901
Priacanthus blochii|BIFZC355-17|IRD BMF-640.2|BOLD:ADB4901
Priacanthus blochii|BIFZC352-17|IRD BMF-260.2|BOLD:ADB4901
Priacanthus blochii|BIFZC356-17|IRD BMF-641.2|BOLD:ADB4901
Priacanthus blochii|BIFZC357-17|IRD BMF-642.2|BOLD:ADB4901
Priacanthus blochii|BIFZC354-17|IRD BMF-639.2|BOLD:ADB4901
Priacanthus macracanthus|BIFZC349-17|IRD BMF-86.2|BOLD:AAC1984
Priacanthus macracanthus|BIFZC348-17|IRD BMF-85.2|BOLD:AAC1984
Priacanthus macracanthus|BIFZC347-17|IRD BMF-84.2|BOLD:AAC1984
Priacanthus macracanthus|BIFZC350-17|IRD BMF-87.2|BOLD:AAC1984
Priacanthus macracanthus|BIFZC351-17|IRD BMF-88.2|BOLD:AAC1984
Priacanthus hamrur|BIFZC340-17|IRD BMF-636.2|BOLD:AAB1643
Priacanthus hamrur|BIFZC341-17|IRD BMF-637.2|BOLD:AAB1643
Priacanthus hamrur|DBMFA532-16|BMF-705.2|BOLD:AAB1643
Priacanthus hamrur|BIFZC342-17|IRD BMF-679.2|BOLD:AAB1643
Priacanthus hamrur|BIFZC345-17|IRD BMF-704.2|BOLD:AAB1643
Priacanthus hamrur|BIFZC343-17|IRD BMF-680.2|BOLD:AAB1643
Pempheris adusta|BIFZC322-17|IRD BMF-358.2|BOLD:AAC1932
Pempheris oualensis|BIFZC318-17|IRD BMF-354.2|BOLD:ACV7285
Pempheris oualensis|BIFZC321-17|IRD BMF-357.2|BOLD:ACV7285
Pempheris oualensis|BIFZC320-17|IRD BMF-356.2|BOLD:ACV7285
Pempheris oualensis|BIFZC319-17|IRD BMF-355.2|BOLD:ACV7285
Neoniphon aurolineatus|BIFZB152-17|IRD BMF-656.2|BOLD:AAC4648
Neoniphon aurolineatus|BIFZB151-17|IRD BMF-572.2|BOLD:AAC4648
Neoniphon aurolineatus|BIFZB153-17|IRD BMF-657.2|BOLD:AAC4648
Neoniphon aurolineatus|BIFZB150-17|IRD BMF-415.2|BOLD:AAC4648
Neoniphon argenteus|BIFZB146-17|IRD BMF-533.2|BOLD:AAH7514
Neoniphon argenteus|BIFZB149-17|IRD BMF-549.2|BOLD:AAH7514
Neoniphon argenteus|BIFZB148-17|IRD BMF-535.2|BOLD:AAH7514

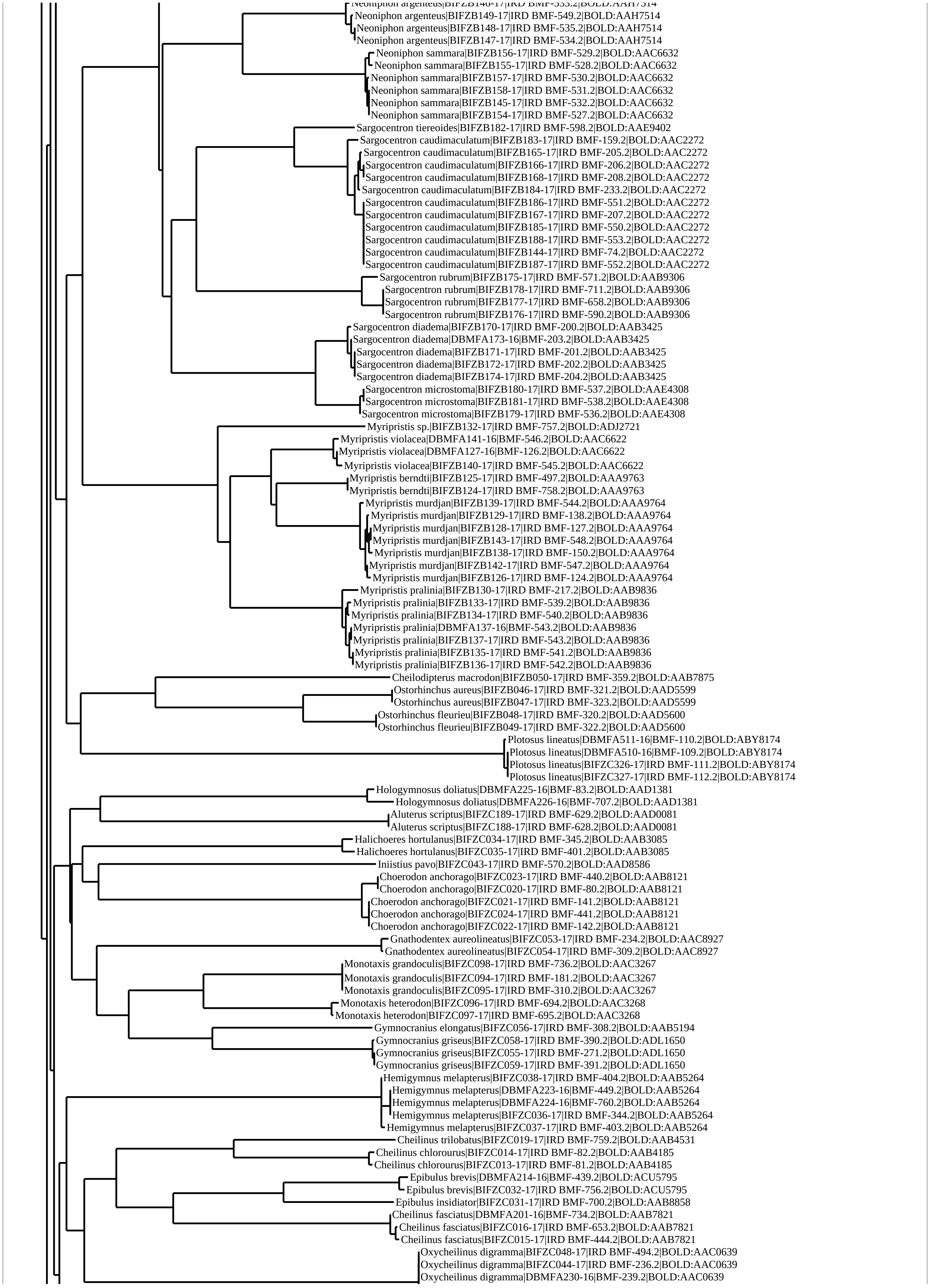
Neoniphon argenteus|BIFZB146-17|IRD BMF-533.2|BOLD:AAH7514
Neoniphon argenteus|BIFZB149-17|IRD BMF-549.2|BOLD:AAH7514
Neoniphon argenteus|BIFZB148-17|IRD BMF-535.2|BOLD:AAH7514
Neoniphon argenteus|BIFZB147-17|IRD BMF-534.2|BOLD:AAH7514
Neoniphon sammara|BIFZB156-17|IRD BMF-529.2|BOLD:AAC6632
Neoniphon sammara|BIFZB155-17|IRD BMF-528.2|BOLD:AAC6632
Neoniphon sammara|BIFZB157-17|IRD BMF-530.2|BOLD:AAC6632
Neoniphon sammara|BIFZB158-17|IRD BMF-531.2|BOLD:AAC6632
Neoniphon sammara|BIFZB145-17|IRD BMF-532.2|BOLD:AAC6632
Neoniphon sammara|BIFZB154-17|IRD BMF-527.2|BOLD:AAC6632
Sargocentron tiereoides|BIFZB182-17|IRD BMF-598.2|BOLD:AAE9402
Sargocentron caudimaculatum|BIFZB183-17|IRD BMF-159.2|BOLD:AAC2272
Sargocentron caudimaculatum|BIFZB165-17|IRD BMF-205.2|BOLD:AAC2272
Sargocentron caudimaculatum|BIFZB166-17|IRD BMF-206.2|BOLD:AAC2272
Sargocentron caudimaculatum|BIFZB168-17|IRD BMF-208.2|BOLD:AAC2272
Sargocentron caudimaculatum|BIFZB184-17|IRD BMF-233.2|BOLD:AAC2272
Sargocentron caudimaculatum|BIFZB186-17|IRD BMF-551.2|BOLD:AAC2272
Sargocentron caudimaculatum|BIFZB167-17|IRD BMF-207.2|BOLD:AAC2272
Sargocentron caudimaculatum|BIFZB185-17|IRD BMF-550.2|BOLD:AAC2272
Sargocentron caudimaculatum|BIFZB188-17|IRD BMF-553.2|BOLD:AAC2272
Sargocentron caudimaculatum|BIFZB144-17|IRD BMF-74.2|BOLD:AAC2272
Sargocentron caudimaculatum|BIFZB187-17|IRD BMF-552.2|BOLD:AAC2272
Sargocentron rubrum|BIFZB175-17|IRD BMF-571.2|BOLD:AAB9306
Sargocentron rubrum|BIFZB178-17|IRD BMF-711.2|BOLD:AAB9306
Sargocentron rubrum|BIFZB177-17|IRD BMF-658.2|BOLD:AAB9306
Sargocentron rubrum|BIFZB176-17|IRD BMF-590.2|BOLD:AAB9306
Sargocentron diadema|BIFZB170-17|IRD BMF-200.2|BOLD:AAB3425
Sargocentron diadema|DBMFA173-16|BMF-203.2|BOLD:AAB3425
Sargocentron diadema|BIFZB171-17|IRD BMF-201.2|BOLD:AAB3425
Sargocentron diadema|BIFZB172-17|IRD BMF-202.2|BOLD:AAB3425
Sargocentron diadema|BIFZB174-17|IRD BMF-204.2|BOLD:AAB3425
Sargocentron microstoma|BIFZB180-17|IRD BMF-537.2|BOLD:AAE4308
Sargocentron microstoma|BIFZB181-17|IRD BMF-538.2|BOLD:AAE4308
Sargocentron microstoma|BIFZB179-17|IRD BMF-536.2|BOLD:AAE4308
Myripristis sp.|BIFZB132-17|IRD BMF-757.2|BOLD:ADJ2721
Myripristis violacea|DBMFA141-16|BMF-546.2|BOLD:AAC6622
Myripristis violacea|DBMFA127-16|BMF-126.2|BOLD:AAC6622
Myripristis violacea|BIFZB140-17|IRD BMF-545.2|BOLD:AAC6622
Myripristis berndti|BIFZB125-17|IRD BMF-497.2|BOLD:AAA9763
Myripristis berndti|BIFZB124-17|IRD BMF-758.2|BOLD:AAA9763
Myripristis murdjan|BIFZB139-17|IRD BMF-544.2|BOLD:AAA9764
Myripristis murdjan|BIFZB129-17|IRD BMF-138.2|BOLD:AAA9764
Myripristis murdjan|BIFZB128-17|IRD BMF-127.2|BOLD:AAA9764
Myripristis murdjan|BIFZB143-17|IRD BMF-548.2|BOLD:AAA9764
Myripristis murdjan|BIFZB138-17|IRD BMF-150.2|BOLD:AAA9764
Myripristis murdjan|BIFZB142-17|IRD BMF-547.2|BOLD:AAA9764
Myripristis murdjan|BIFZB126-17|IRD BMF-124.2|BOLD:AAA9764
Myripristis pralinia|BIFZB130-17|IRD BMF-217.2|BOLD:AAB9836
Myripristis pralinia|BIFZB133-17|IRD BMF-539.2|BOLD:AAB9836
Myripristis pralinia|BIFZB134-17|IRD BMF-540.2|BOLD:AAB9836
Myripristis pralinia|DBMFA137-16|BMF-543.2|BOLD:AAB9836
Myripristis pralinia|BIFZB137-17|IRD BMF-543.2|BOLD:AAB9836
Myripristis pralinia|BIFZB135-17|IRD BMF-541.2|BOLD:AAB9836
Myripristis pralinia|BIFZB136-17|IRD BMF-542.2|BOLD:AAB9836
Cheilodipterus macrodon|BIFZB050-17|IRD BMF-359.2|BOLD:AAB7875
Ostorhinchus aureus|BIFZB046-17|IRD BMF-321.2|BOLD:AAD5599
Ostorhinchus aureus|BIFZB047-17|IRD BMF-323.2|BOLD:AAD5599
Ostorhinchus fleurieu|BIFZB048-17|IRD BMF-320.2|BOLD:AAD5600
Ostorhinchus fleurieu|BIFZB049-17|IRD BMF-322.2|BOLD:AAD5600
Plotosus lineatus|DBMFA511-16|BMF-110.2|BOLD:ABY8174
Plotosus lineatus|DBMFA510-16|BMF-109.2|BOLD:ABY8174
Plotosus lineatus|BIFZC326-17|IRD BMF-111.2|BOLD:ABY8174
Plotosus lineatus|BIFZC327-17|IRD BMF-112.2|BOLD:ABY8174
Hologymnosus doliatus|DBMFA225-16|BMF-83.2|BOLD:AAD1381
Hologymnosus doliatus|DBMFA226-16|BMF-707.2|BOLD:AAD1381
Aluterus scriptus|BIFZC189-17|IRD BMF-629.2|BOLD:AAD0081
Aluterus scriptus|BIFZC188-17|IRD BMF-628.2|BOLD:AAD0081
Halichoeres hortulanus|BIFZC034-17|IRD BMF-345.2|BOLD:AAB3085
Halichoeres hortulanus|BIFZC035-17|IRD BMF-401.2|BOLD:AAB3085
Iniistius pavo|BIFZC043-17|IRD BMF-570.2|BOLD:AAD8586
Choerodon anchorago|BIFZC023-17|IRD BMF-440.2|BOLD:AAB8121
Choerodon anchorago|BIFZC020-17|IRD BMF-80.2|BOLD:AAB8121
Choerodon anchorago|BIFZC021-17|IRD BMF-141.2|BOLD:AAB8121
Choerodon anchorago|BIFZC024-17|IRD BMF-441.2|BOLD:AAB8121
Choerodon anchorago|BIFZC022-17|IRD BMF-142.2|BOLD:AAB8121
Gnathodentex aureolineatus|BIFZC053-17|IRD BMF-234.2|BOLD:AAC8927
Gnathodentex aureolineatus|BIFZC054-17|IRD BMF-309.2|BOLD:AAC8927
Monotaxis grandoculis|BIFZC098-17|IRD BMF-736.2|BOLD:AAC3267
Monotaxis grandoculis|BIFZC094-17|IRD BMF-181.2|BOLD:AAC3267
Monotaxis grandoculis|BIFZC095-17|IRD BMF-310.2|BOLD:AAC3267
Monotaxis heterodon|BIFZC096-17|IRD BMF-694.2|BOLD:AAC3268
Monotaxis heterodon|BIFZC097-17|IRD BMF-695.2|BOLD:AAC3268
Gymnocranius elongatus|BIFZC056-17|IRD BMF-308.2|BOLD:AAB5194
Gymnocranius griseus|BIFZC058-17|IRD BMF-390.2|BOLD:ADL1650
Gymnocranius griseus|BIFZC055-17|IRD BMF-271.2|BOLD:ADL1650
Gymnocranius griseus|BIFZC059-17|IRD BMF-391.2|BOLD:ADL1650
Hemigymnus melapterus|BIFZC038-17|IRD BMF-404.2|BOLD:AAB5264
Hemigymnus melapterus|DBMFA223-16|BMF-449.2|BOLD:AAB5264
Hemigymnus melapterus|DBMFA224-16|BMF-760.2|BOLD:AAB5264
Hemigymnus melapterus|BIFZC036-17|IRD BMF-344.2|BOLD:AAB5264
Hemigymnus melapterus|BIFZC037-17|IRD BMF-403.2|BOLD:AAB5264
Cheilinus trilobatus|BIFZC019-17|IRD BMF-759.2|BOLD:AAB4531
Cheilinus chlorourus|BIFZC014-17|IRD BMF-82.2|BOLD:AAB4185
Cheilinus chlorourus|BIFZC013-17|IRD BMF-81.2|BOLD:AAB4185
Epibulus brevis|DBMFA214-16|BMF-439.2|BOLD:ACU5795
Epibulus brevis|BIFZC032-17|IRD BMF-756.2|BOLD:ACU5795
Epibulus insidiator|BIFZC031-17|IRD BMF-700.2|BOLD:AAB8858
Cheilinus fasciatus|DBMFA201-16|BMF-734.2|BOLD:AAB7821
Cheilinus fasciatus|BIFZC016-17|IRD BMF-653.2|BOLD:AAB7821
Cheilinus fasciatus|BIFZC015-17|IRD BMF-444.2|BOLD:AAB7821
Oxycheilinus digramma|BIFZC048-17|IRD BMF-494.2|BOLD:AAC0639
Oxycheilinus digramma|BIFZC044-17|IRD BMF-236.2|BOLD:AAC0639
Oxycheilinus digramma|DBMFA230-16|BMF-239.2|BOLD:AAC0639

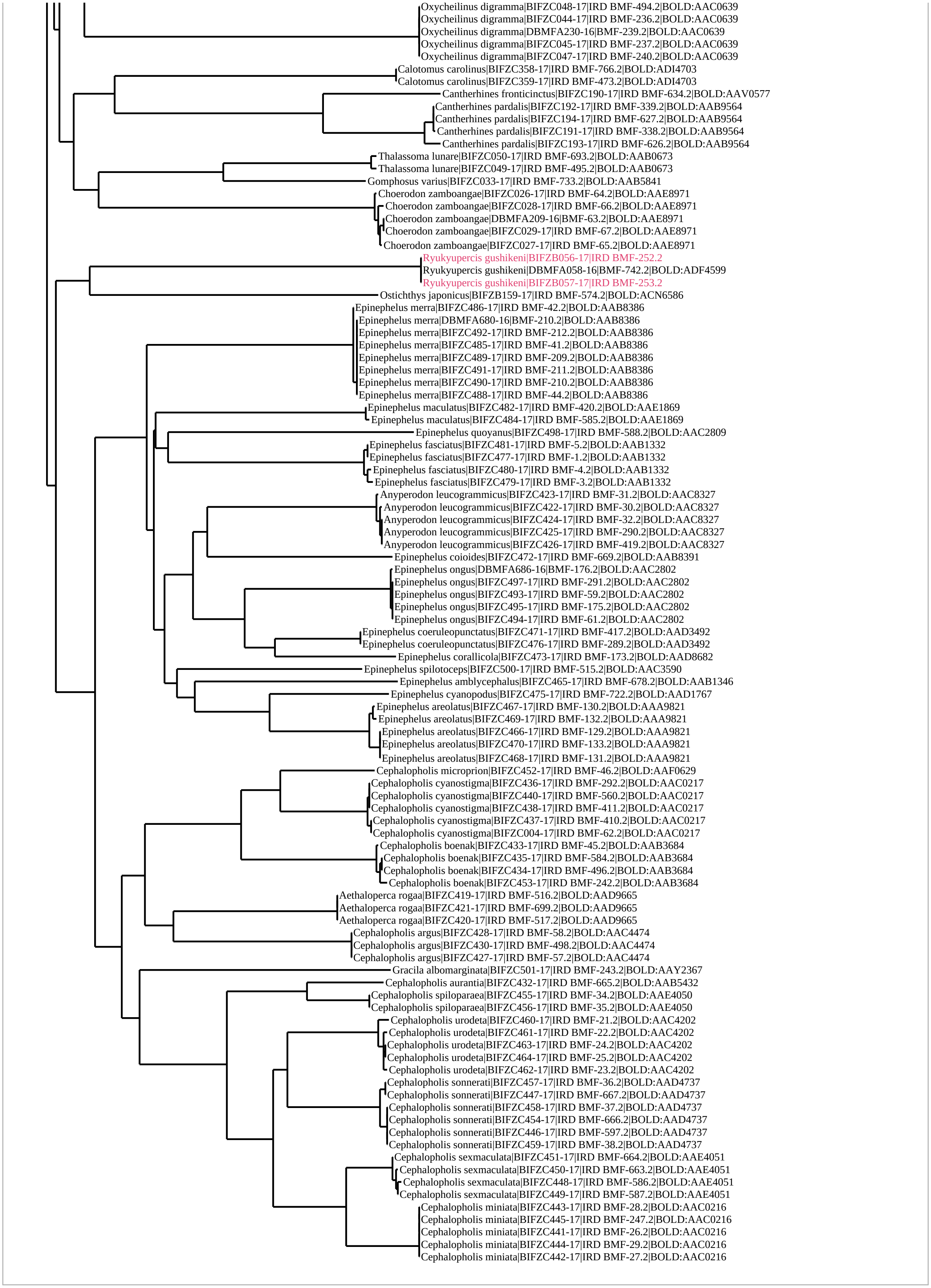
Oxycheilinus digramma|BIFZC048-17|IRD BMF-494.2|BOLD:AAC0639
Oxycheilinus digramma|BIFZC044-17|IRD BMF-236.2|BOLD:AAC0639
Oxycheilinus digramma|DBMFA230-16|BMF-239.2|BOLD:AAC0639
Oxycheilinus digramma|BIFZC045-17|IRD BMF-237.2|BOLD:AAC0639
Oxycheilinus digramma|BIFZC047-17|IRD BMF-240.2|BOLD:AAC0639
Calotomus carolinus|BIFZC358-17|IRD BMF-766.2|BOLD:ADI4703
Calotomus carolinus|BIFZC359-17|IRD BMF-473.2|BOLD:ADI4703
Cantherhines fronticinctus|BIFZC190-17|IRD BMF-634.2|BOLD:AAV0577
Cantherhines pardalis|BIFZC192-17|IRD BMF-339.2|BOLD:AAB9564
Cantherhines pardalis|BIFZC194-17|IRD BMF-627.2|BOLD:AAB9564
Cantherhines pardalis|BIFZC191-17|IRD BMF-338.2|BOLD:AAB9564
Cantherhines pardalis|BIFZC193-17|IRD BMF-626.2|BOLD:AAB9564
Thalassoma lunare|BIFZC050-17|IRD BMF-693.2|BOLD:AAB0673
Thalassoma lunare|BIFZC049-17|IRD BMF-495.2|BOLD:AAB0673
Gomphosus varius|BIFZC033-17|IRD BMF-733.2|BOLD:AAB5841
Choerodon zamboangae|BIFZC026-17|IRD BMF-64.2|BOLD:AAE8971
Choerodon zamboangae|BIFZC028-17|IRD BMF-66.2|BOLD:AAE8971
Choerodon zamboangae|DBMFA209-16|BMF-63.2|BOLD:AAE8971
Choerodon zamboangae|BIFZC029-17|IRD BMF-67.2|BOLD:AAE8971
Choerodon zamboangae|BIFZC027-17|IRD BMF-65.2|BOLD:AAE8971
Ryukyupercis gushikeni|BIFZB056-17|IRD BMF-252.2
Ryukyupercis gushikeni|DBMFA058-16|BMF-742.2|BOLD:ADF4599
Ryukyupercis gushikeni|BIFZB057-17|IRD BMF-253.2
Ostichthys japonicus|BIFZB159-17|IRD BMF-574.2|BOLD:ACN6586
Epinephelus merra|BIFZC486-17|IRD BMF-42.2|BOLD:AAB8386
Epinephelus merra|DBMFA680-16|BMF-210.2|BOLD:AAB8386
Epinephelus merra|BIFZC492-17|IRD BMF-212.2|BOLD:AAB8386
Epinephelus merra|BIFZC485-17|IRD BMF-41.2|BOLD:AAB8386
Epinephelus merra|BIFZC489-17|IRD BMF-209.2|BOLD:AAB8386
Epinephelus merra|BIFZC491-17|IRD BMF-211.2|BOLD:AAB8386
Epinephelus merra|BIFZC490-17|IRD BMF-210.2|BOLD:AAB8386
Epinephelus merra|BIFZC488-17|IRD BMF-44.2|BOLD:AAB8386
Epinephelus maculatus|BIFZC482-17|IRD BMF-420.2|BOLD:AAE1869
Epinephelus maculatus|BIFZC484-17|IRD BMF-585.2|BOLD:AAE1869
Epinephelus quoyanus|BIFZC498-17|IRD BMF-588.2|BOLD:AAC2809
Epinephelus fasciatus|BIFZC481-17|IRD BMF-5.2|BOLD:AAB1332
Epinephelus fasciatus|BIFZC477-17|IRD BMF-1.2|BOLD:AAB1332
Epinephelus fasciatus|BIFZC480-17|IRD BMF-4.2|BOLD:AAB1332
Epinephelus fasciatus|BIFZC479-17|IRD BMF-3.2|BOLD:AAB1332
Anyperodon leucogrammicus|BIFZC423-17|IRD BMF-31.2|BOLD:AAC8327
Anyperodon leucogrammicus|BIFZC422-17|IRD BMF-30.2|BOLD:AAC8327
Anyperodon leucogrammicus|BIFZC424-17|IRD BMF-32.2|BOLD:AAC8327
Anyperodon leucogrammicus|BIFZC425-17|IRD BMF-290.2|BOLD:AAC8327
Anyperodon leucogrammicus|BIFZC426-17|IRD BMF-419.2|BOLD:AAC8327
Epinephelus coioides|BIFZC472-17|IRD BMF-669.2|BOLD:AAB8391
Epinephelus ongus|DBMFA686-16|BMF-176.2|BOLD:AAC2802
Epinephelus ongus|BIFZC497-17|IRD BMF-291.2|BOLD:AAC2802
Epinephelus ongus|BIFZC493-17|IRD BMF-59.2|BOLD:AAC2802
Epinephelus ongus|BIFZC495-17|IRD BMF-175.2|BOLD:AAC2802
Epinephelus ongus|BIFZC494-17|IRD BMF-61.2|BOLD:AAC2802
Epinephelus coeruleopunctatus|BIFZC471-17|IRD BMF-417.2|BOLD:AAD3492
Epinephelus coeruleopunctatus|BIFZC476-17|IRD BMF-289.2|BOLD:AAD3492
Epinephelus corallicola|BIFZC473-17|IRD BMF-173.2|BOLD:AAD8682
Epinephelus spilotoceps|BIFZC500-17|IRD BMF-515.2|BOLD:AAC3590
Epinephelus amblycephalus|BIFZC465-17|IRD BMF-678.2|BOLD:AAB1346
Epinephelus cyanopodus|BIFZC475-17|IRD BMF-722.2|BOLD:AAD1767
Epinephelus areolatus|BIFZC467-17|IRD BMF-130.2|BOLD:AAA9821
Epinephelus areolatus|BIFZC469-17|IRD BMF-132.2|BOLD:AAA9821
Epinephelus areolatus|BIFZC466-17|IRD BMF-129.2|BOLD:AAA9821
Epinephelus areolatus|BIFZC470-17|IRD BMF-133.2|BOLD:AAA9821
Epinephelus areolatus|BIFZC468-17|IRD BMF-131.2|BOLD:AAA9821
Cephalopholis microprion|BIFZC452-17|IRD BMF-46.2|BOLD:AAF0629
Cephalopholis cyanostigma|BIFZC436-17|IRD BMF-292.2|BOLD:AAC0217
Cephalopholis cyanostigma|BIFZC440-17|IRD BMF-560.2|BOLD:AAC0217
Cephalopholis cyanostigma|BIFZC438-17|IRD BMF-411.2|BOLD:AAC0217
Cephalopholis cyanostigma|BIFZC437-17|IRD BMF-410.2|BOLD:AAC0217
Cephalopholis cyanostigma|BIFZC004-17|IRD BMF-62.2|BOLD:AAC0217
Cephalopholis boenak|BIFZC433-17|IRD BMF-45.2|BOLD:AAB3684
Cephalopholis boenak|BIFZC435-17|IRD BMF-584.2|BOLD:AAB3684
Cephalopholis boenak|BIFZC434-17|IRD BMF-496.2|BOLD:AAB3684
Cephalopholis boenak|BIFZC453-17|IRD BMF-242.2|BOLD:AAB3684
Aethaloperca rogaa|BIFZC419-17|IRD BMF-516.2|BOLD:AAD9665
Aethaloperca rogaa|BIFZC421-17|IRD BMF-699.2|BOLD:AAD9665
Aethaloperca rogaa|BIFZC420-17|IRD BMF-517.2|BOLD:AAD9665
Cephalopholis argus|BIFZC428-17|IRD BMF-58.2|BOLD:AAC4474
Cephalopholis argus|BIFZC430-17|IRD BMF-498.2|BOLD:AAC4474
Cephalopholis argus|BIFZC427-17|IRD BMF-57.2|BOLD:AAC4474
Gracila albomarginata|BIFZC501-17|IRD BMF-243.2|BOLD:AAY2367
Cephalopholis aurantia|BIFZC432-17|IRD BMF-665.2|BOLD:AAB5432
Cephalopholis spiloparaea|BIFZC455-17|IRD BMF-34.2|BOLD:AAE4050
Cephalopholis spiloparaea|BIFZC456-17|IRD BMF-35.2|BOLD:AAE4050
Cephalopholis urodeta|BIFZC460-17|IRD BMF-21.2|BOLD:AAC4202
Cephalopholis urodeta|BIFZC461-17|IRD BMF-22.2|BOLD:AAC4202
Cephalopholis urodeta|BIFZC463-17|IRD BMF-24.2|BOLD:AAC4202
Cephalopholis urodeta|BIFZC464-17|IRD BMF-25.2|BOLD:AAC4202
Cephalopholis urodeta|BIFZC462-17|IRD BMF-23.2|BOLD:AAC4202
Cephalopholis sonnerati|BIFZC457-17|IRD BMF-36.2|BOLD:AAD4737
Cephalopholis sonnerati|BIFZC447-17|IRD BMF-667.2|BOLD:AAD4737
Cephalopholis sonnerati|BIFZC458-17|IRD BMF-37.2|BOLD:AAD4737
Cephalopholis sonnerati|BIFZC454-17|IRD BMF-666.2|BOLD:AAD4737
Cephalopholis sonnerati|BIFZC446-17|IRD BMF-597.2|BOLD:AAD4737
Cephalopholis sonnerati|BIFZC459-17|IRD BMF-38.2|BOLD:AAD4737
Cephalopholis sexmaculata|BIFZC451-17|IRD BMF-664.2|BOLD:AAE4051
Cephalopholis sexmaculata|BIFZC450-17|IRD BMF-663.2|BOLD:AAE4051
Cephalopholis sexmaculata|BIFZC448-17|IRD BMF-586.2|BOLD:AAE4051
Cephalopholis sexmaculata|BIFZC449-17|IRD BMF-587.2|BOLD:AAE4051
Cephalopholis miniata|BIFZC443-17|IRD BMF-28.2|BOLD:AAC0216
Cephalopholis miniata|BIFZC445-17|IRD BMF-247.2|BOLD:AAC0216
Cephalopholis miniata|BIFZC441-17|IRD BMF-26.2|BOLD:AAC0216
Cephalopholis miniata|BIFZC444-17|IRD BMF-29.2|BOLD:AAC0216
Cephalopholis miniata|BIFZC442-17|IRD BMF-27.2|BOLD:AAC0216
